# Agent‐based modeling of the effects of forest dynamics, selective logging, and fragment size on epiphyte communities

**DOI:** 10.1002/ece3.7255

**Published:** 2021-02-28

**Authors:** Gunnar Petter, Gerhard Zotz, Holger Kreft, Juliano Sarmento Cabral

**Affiliations:** ^1^ Biodiversity, Macroecology & Biogeography University of Göttingen Göttingen Germany; ^2^ Department of Environmental Systems Science Institute of Terrestrial Ecosystems ETH Zurich Zurich Switzerland; ^3^ Functional Ecology Group Institute of Biology and Environmental Sciences Carl von Ossietzky University Oldenburg Oldenburg Germany; ^4^ Smithsonian Tropical Research Institute Balboa Panamá; ^5^ Centre of Biodiversity and Sustainable Land Use (CBL) University of Göttingen Germany; ^6^ Ecosystem Modeling Center for Computational and Theoretical Biology (CCTB) University of Würzburg Würzburg Germany

**Keywords:** canopy dynamics, community assembly, demography, epiphyte assemblages, epiphyte interactions, forest, individual‐based model, long‐term dynamics, vascular epiphytes

## Abstract

1. Forest canopies play a crucial role in structuring communities of vascular epiphytes by providing substrate for colonization, by locally varying microclimate, and by causing epiphyte mortality due to branch or tree fall. However, as field studies in the three‐dimensional habitat of epiphytes are generally challenging, our understanding of how forest structure and dynamics influence the structure and dynamics of epiphyte communities is scarce.
2. Mechanistic models can improve our understanding of epiphyte community dynamics. We present such a model that couples dispersal, growth, and mortality of individual epiphytes with substrate dynamics, obtained from a three‐dimensional functional–structural forest model, allowing the study of forest–epiphyte interactions. After validating the epiphyte model with independent field data, we performed several theoretical simulation experiments to assess how (a) differences in natural forest dynamics, (b) selective logging, and (c) forest fragmentation could influence the long‐term dynamics of epiphyte communities.
3. The proportion of arboreal substrate occupied by epiphytes (i.e., saturation level) was tightly linked with forest dynamics and increased with decreasing forest turnover rates. While species richness was, in general, negatively correlated with forest turnover rates, low species numbers in forests with very‐low‐turnover rates were due to competitive exclusion when epiphyte communities became saturated. Logging had a negative impact on epiphyte communities, potentially leading to a near‐complete extirpation of epiphytes when the simulated target diameters fell below a threshold. Fragment size had no effect on epiphyte abundance and saturation level but correlated positively with species numbers.
4. Synthesis: The presented model is a first step toward studying the dynamic forest–epiphyte interactions in an agent‐based modeling framework. Our study suggests forest dynamics as key factor in controlling epiphyte communities. Thus, both natural and human‐induced changes in forest dynamics, for example, increased mortality rates or the loss of large trees, pose challenges for epiphyte conservation.

## Graphical abstract

Field studies in the three‐dimensional habitat of epiphytes are generally challenging, and our understanding of how forests influence epiphyte communities is thus scarce. By developing and applying an agent‐based model, we were able to analyze forest–epiphyte interactions and demonstrate how forest dynamics can control epiphyte communities.

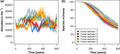

## 1 INTRODUCTION

Vascular epiphytes are arboreal organisms that germinate and grow on other plants, usually trees, using this previously unexploited spatial resource (Zotz, 2016). As epiphytes do never have contact to forest soil, they have to cope with low and irregular supply of water and nutrients from atmospheric inputs, litter or canopy soils (Reynolds & Hunter, 2004; Wania et al., 2002). Epiphytes are key components of tropical ecosystems, where they contribute substantially to local plant diversity (Kelly et al., 2004), play an important role for water supply and nutrient cycling by retaining precipitation in the canopy and by contributing to the formation of canopy soil and ground litter (Stanton et al., 2014), and provide microhabitats and food for arboreal animals (Stuntz et al., 2002; Yanoviak et al., 2007).

At large spatial scales, the diversity, abundance, and structure of epiphyte communities are strongly influenced by environmental conditions, in particular by water availability (e.g., Kreft et al., 2004). At smaller spatial scales, forest structure and dynamics are assumed to be important drivers of community assembly (Ding et al., 2016). On the one hand, forests indirectly influence epiphyte communities by modifying the spatial distribution of environmental factors. Forest canopies are typically characterized by pronounced vertical gradients in abiotic factors, for example, light or humidity, which influence the vertical distribution of epiphytes (e.g., Petter et al., 2016; Zotz, 2007). On the other hand, forests directly influence the demographic processes of epiphytes as they provide essential substrate for colonization. Since new substrate is continuously generated by tree growth and lost via branch or tree fall, forests influence both establishment and mortality of epiphytes. Few studies deal with the direct effects of tree growth and forest dynamics on epiphytes. For instance, Hietz (1997) and Sarmento Cabral et al. (2015) reported high mortality rates due to branch fall, and Ding et al. (2016) found that forest structure may explain a similar proportion of variance in abundance and species richness as humidity. These studies highlight the fundamental importance of forest structure and dynamics for understanding the structure and dynamics of epiphyte communities. Currently, our knowledge on the link between forest and epiphyte dynamics is very limited, but such knowledge is urgently needed given the increasing human impact on forest dynamics via, for example, selective logging, forest fragmentation, and edge effects (Lewis et al., 2015). Additionally, climate change is altering forest dynamics, with increasing forest background mortality rates (e.g., Allen et al., 2010; Phillips et al., 2008) and an increased mortality risk of large trees during droughts (Ryan, 2015). To conserve epiphytes, we need to understand the feedbacks of such changes on epiphyte communities to plan the most adequate mitigation policies.

The limited availability of data on the long‐term dynamics of epiphyte populations and communities hampers the development of general theory on the relationship between forest and epiphyte dynamics. Difficulties in accessing the canopy for sampling and monitoring epiphytes in their three‐dimensional habitat demand elevated funding and work effort. Consequently, studies on the composition, structure, and, in particular, the dynamics of epiphyte populations and communities are scarce compared to the numerous floristic works on epiphytes. Mondragón et al. (2015) reviewed population ecology studies of epiphytic angiosperms: Population matrix analyses are available for only 30 species in two families (bromeliads, orchids). Regarding the temporal dynamics of epiphyte communities, only three repeated plot‐scale censuses (1 ha plot in Venezuela: Schmit‐Neuerburg, 2002; 0.4 ha plot in Panama: first census by Zotz & Schultz, 2008, second census by G. Mendieta‐Leiva, K. Wagner & G. Zotz, unpublished data; c. 80 1‐ha plots in pastures by Poltz & Zotz, 2011, and by Einzmann & Zotz, 2017) and two studies assessing temporal changes on specific host tree species (*Socratea exorrhiza*: Laube & Zotz, 2006; *Annona glabra*: Zotz et al., 1999) exist. These studies provide valuable insights into the temporal dynamics of epiphyte populations and communities, but they do not allow inference about the relationship between forest and epiphyte dynamics, as forest dynamics, as possible codeterminants, were not recorded. Whenever data availability is a limiting factor, mechanistic models can help to improve ecological understanding and, in the case of epiphytes, unravel the complex link between forests and epiphytes.

Here, we present an individual‐based model that simulates the dynamics of epiphyte communities in three‐dimensional space. In this model, epiphyte community dynamics are determined by two main components, namely the species‐specific functional traits of the epiphytes and the forest. The traits of the epiphyte species in the community determine how they grow, reproduce, interact, and die. The forest and its dynamics provide the physical space and environmental conditions for the epiphytes, for example, by affecting within‐canopy light conditions and substrate availability. Both light and substrate availability vary dynamically due to tree or branch fall and regeneration. This model setup allows separating the effects of the intrinsic, trait‐driven epiphyte community dynamics from the effects of forest dynamics by simulating the same epiphyte communities on different forest systems. Following this basic idea, and after validating the epiphyte model with spatial data of epiphytes, we have set up several theoretical simulation experiments in which we used information on three‐dimensional forest structure and dynamics as input data. We generated these data with a three‐dimensional forest model that simulates structural growth, establishment, and mortality of trees and branches at the stand scale. The forests used in the simulation experiments differed regarding their dynamics (biomass turnover) and the size of the forest fragment. In addition, we generated theoretical logging scenarios in which trees were removed once they reached different target diameter. These simulations showcase a variety of experiments that can be performed with this first spatially explicit and niche‐based epiphyte model and indicate how important forest dynamics can be for the diversity and abundance of vascular epiphyte communities.

## 2 MATERIALS AND METHODS

Our model is individual‐based, operates in three‐dimensional space, and uses information on the spatial structure and temporal changes of forests at a high resolution (1 m^3^) as input data. Such data could be obtained from ground or airborne LiDAR scans or from statistical and process‐based models. Here, we used a previously developed three‐dimensional functional–structural forest model that realistically reproduced the structure and long‐term dynamics of tropical forests as data source (Petter et al., 2020; Appendix S1).

### 2.1 Model description

The model description follows the ODD (**O**verview, **D**esign concepts, **D**etails) protocol, which was proposed as standard protocol to describe agent‐based models (Grimm et al., 2006, 2010). In the following, the overview part of the model description is provided. The full model description is available in Appendix S2. The MATLAB (2016) source code is available at https://github.com/julianoscabral/MoDVE.

#### 2.1.1 Purpose

The main purpose of this model is to analyze the influence of forest dynamics on the structure and dynamics of vascular epiphyte communities. Vascular epiphytes germinate and grow on trees. Hence, their fate is connected to the dynamics of their host trees, which grow and create new substrate, but also shed branches and ultimately fall and die (Sarmento Cabral et al., 2015; Spruch et al., 2019; Taylor & Burns, 2015). Driven by differences in the natural environment or by human interventions, forest dynamics can vary substantially (Brown et al., 2004; Quesada et al., 2012; Wright, 2005). We studied the impact of such variations on epiphyte communities.

#### 2.1.2 Entities, state variables, and scales

**FIGURE 1:**
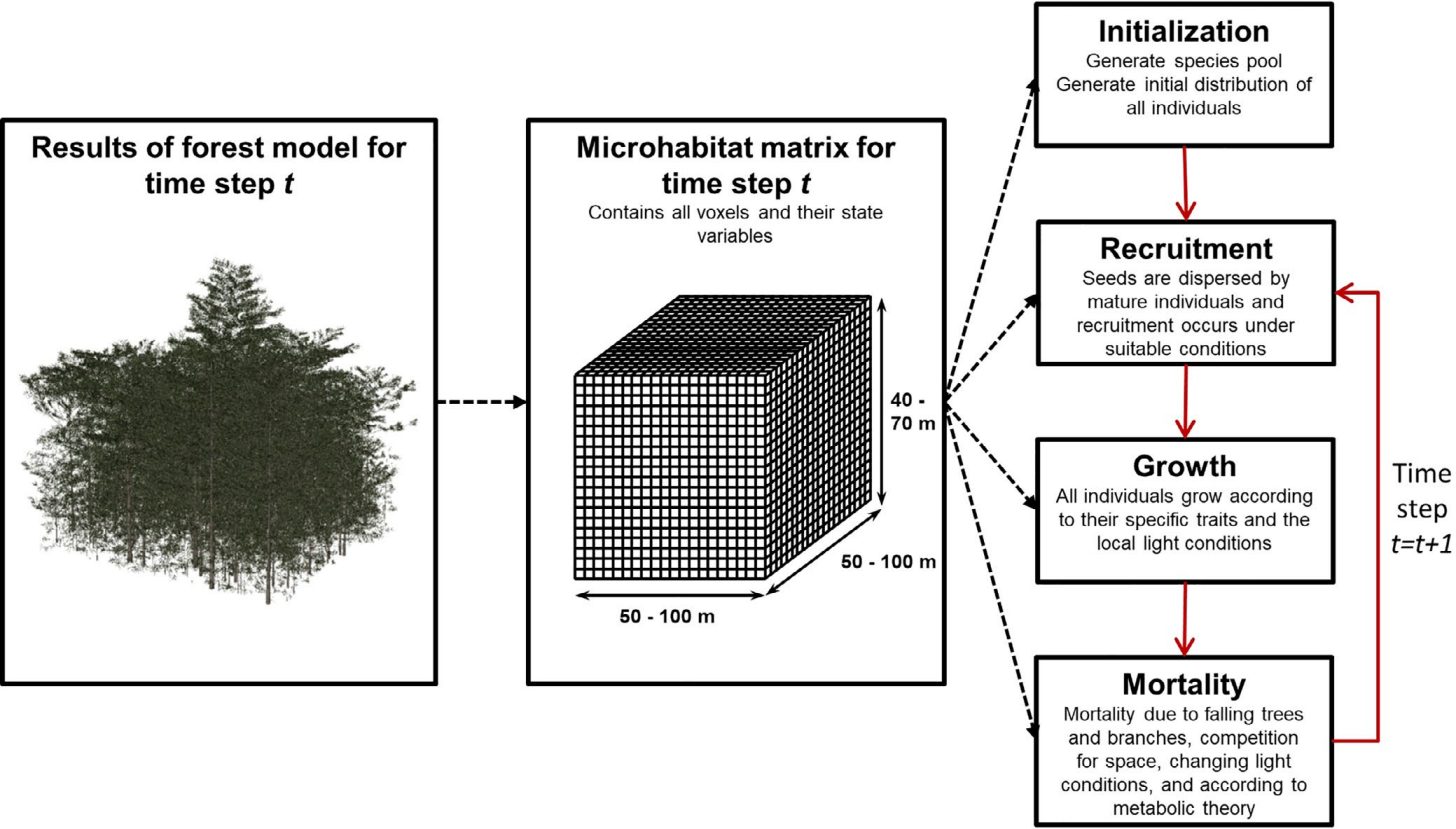
Flowchart of the coupled forest–epiphyte model. Based on dynamic three‐dimensional input data from a functional–structural forest model (Petter et al., 2020), a microhabitat matrix characterizing the epiphytic habitat at each time step is generated. To this end, the simulated spatial distribution of leaf area, branches, and trunks for each annual time step (left panel) is used to calculate the light distribution, total substrate area and relative annual change of substrate area for each 1 m^3^‐voxel in the microhabitat matrix (middle panel), which ultimately influences the initialization and all three submodels of the epiphyte model (right panel)

The epiphyte model is three‐dimensional and voxel‐based, and its spatial extent depends on the spatial dimensions of the input forest data. Here, forests cover an area of 0.25 to 1 hectare and have a canopy height of max. 50 m. The model space is subdivided into voxels of 1 m^3^, whose state variables characterize three key environmental conditions: i) light intensity, ii) total area of arboreal substrate, and iii) relative loss of substrate area (Table 1). We acknowledge that there are other abiotic factors varying within a forest. While some of these, such as humidity, vary in concert with light intensity (Wagner et al., 2013) and are thus included implicitly, others like bark texture (substrate quality) are ignored at this model stage. The model proceeds in annual time steps and the state variables of the voxels are updated each year according to the input data (Figure 1). Individual epiphytes are the ecological entities whose growth, reproduction, and mortality are simulated as functions of their ecological traits and of the environmental conditions in the voxels. The state variables and traits of epiphytes are summarized in Table 1.

**TABLE 1:** State variables and species‐specific traits. The demographic processes and the state variables of individual epiphytes are influenced by the state variables of the voxels (i.e., environmental conditions) and by the specific traits of each species to which an individual epiphyte belongs

Symbol	Description	Unit	Type
*A*	Age of epiphyte	year	State variable (epiphyte)
*E*_X_, *E*_Y_, *E*_Z_	Position of epiphyte in model space in X, Y, Z direction	m	State variable (epiphyte)
*ID*_Ind_	Identifier of epiphyte individuals	‐	State variable (epiphyte)
*ID*_Sp_	Identifier of epiphyte species	‐	State variable (epiphyte)
*M*	Mass of epiphyte	g	State variable (epiphyte)
*I*	Light intensity	μmol/m^2^ s^−1^	State variable (voxel)
S*_B_*	Total surface area of arboreal substrate	m^2^	State variable (voxel)
*S*_Loss_	Percentage annual surface area change in voxel	%	State variable (voxel)
*V*_X_, *V*_Y_, *V*_Z_	Position of voxel in model space in X, Y, Z direction	m	State variable (voxel)
*A*_Mat_	Age at maturity	year	Species‐specific trait
*D*_K_	Dispersal ability—factor B in negative exponential function	–	Species‐specific trait
*D*_KAs_	Dispersal kernel asymmetry	–	Species‐specific trait
*I*_A_, *I*_B_, *I*_C_	Parameters A, B, C of parabolic light response curve	–	Species‐specific trait
*I*_Min_, *I*_Max_, *I*_Opt_	Minimum, maximum, optimum light intensity for survival	μmol/m^2^ s^−1^	Species‐specific trait
*K*	Growth rate (von Bertalanffy growth)	a^−1^	Species‐specific trait
*M*_Mat_	Mass at maturity	g	Species‐specific trait
*M*_Max_	Maximum mass	g	Species‐specific trait
*n*_RPot_	Average potential number of recruits per individual	‐	Species‐specific trait

#### 2.1.3 Process overview and scheduling

Based on the forest input data, three‐dimensional microhabitat matrices containing the state variables of all voxels are calculated for each annual time step (Figure 1). The first microhabitat matrix is used to initialize the distribution of epiphytes (see Appendix S2 for more details). After initialization, recruitment, growth, and mortality of each individual are simulated successively at each time step, as shortly described below.

*Recruitment*: Mature epiphytes reproduce, with the number of new recruits determined by the species‐specific fecundity (*n*_RPot_, Table 1), the species‐specific dispersal kernel (*D*_K_ and *D*_KAs_, Table 1), and the surrounding substrate area of trees and branches. The dispersal probability decreases with distance and the narrower the dispersal kernel, the more likely recruits will establish within the immediate proximity of the mature plant. The actual number of new recruits is calculated based on Poisson random values (see Appendix S2 for details). Clonal vegetative growth is not simulated in the current model version.

*Growth*: Growth of each individual is simulated as a function of its mass and the light conditions in the specific voxel. From the several possible plant growth functions (Paine et al., 2012), we opted for the von Bertalanffy function to achieve generality across species and to maintain a low number of parameters. It describes reasonably well the few known growth trajectories of epiphytes (e.g., Schmidt & Zotz, 2002), and it has only one parameter which can be derived from three species‐specific traits (see Appendix S2 for equations).

*Mortality*: Individuals die when the light conditions are outside the species‐specific light niche, which can occur when the forest structure changes (mortality due to changing environmental conditions). If several individuals occupy the same voxel and their total space requirement exceeds the available surface area, smaller individuals will be outcompeted by larger ones. Mortality also occurs when an individual is alone in a voxel but grows beyond the physical space present in the voxel. This simplification ignores that an individual could grow above the branch or into neighboring voxels, but correctly reflects the possibility that a plant can fall due to its own weight or due to mechanical stress from wind, rain, or animal movement. This way we address the findings of previous studies reporting large individuals falling with a higher frequency off branches and trunks (Sarmento Cabral et al., 2015). The latter two mortality processes summarize resource limitation and competition. Furthermore, individuals may die due to branch or tree fall, with the relative surface loss in a voxel defining the probability of mortality. Additionally, body mass‐dependent mortality probabilities following the quarter‐power law of the metabolic theory of ecology (Brown et al., 2004) account for mortality causes not explicitly simulated (e.g., desiccation or pathogens). Mortality probabilities are used to draw individual deaths from a random Binomial distribution.

After this final step, the age of surviving epiphytes is updated and the model proceeds to the next time step.

### 2.2 Model calibration and validation

Before conducting simulation experiments, we tested whether the model reproduced the long‐term dynamics and structure of epiphyte communities. Although limited knowledge on long‐term dynamics of epiphyte communities hampers model parameterization and evaluation, there are few studies providing information on specific aspects of community dynamics, for example, short‐term community dynamics (Einzmann & Zotz, 2017; Schmit‐Neuerburg, 2002; Zotz et al., 2005) or annual mortality rates (Hietz, 1997; Sarmento Cabral et al., 2015). In contrast, the structure of epiphyte communities is better known. We had access to two datasets from Panama and Ecuador in which the three‐dimensional position and species identity of each epiphytic individual were identified (Panama: 0.4 ha; Ecuador: 0.1 ha). In these datasets, the epiphyte communities showed remarkable similarities in their structure, specifically regarding the vertical distribution of individuals, the vertical stratification of species, and their size and rank abundance distribution. The above‐mentioned information on community dynamics and the datasets from Panama and Ecuador form the basis for the model calibration process (described in the following paragraphs). This means, in an appropriate model the simulated epiphyte community should be in a long‐term dynamic equilibrium state, the average mortality rates should be in the reported ranges, and the community structure should be consistent with the observations from Panama and Ecuador. The available information was collected at different places and at different times, and our aim was to reproduce the general ranges and patterns observed.

To calibrate the epiphyte model, we first determined the reference forest that provided input data for the epiphyte model. We selected a 50 × 50 m forest plot that was simulated with a three‐dimensional forest model and reproduced the structure and long‐term dynamics of a typical Neotropical lowland rainforest (from now on referred as “reference forest”—see Appendix S1 for details on validation). This forest was in dynamic equilibrium after 200 years, and we selected forest dynamics from years 200–800 as input for the epiphyte model.

To assess the epiphyte model, we then initialized unstructured epiphyte communities with equal numbers of individuals (100 individuals) for all species of a species set (100 species), reflecting a realistic density of 40,000 individuals per hectare (Zotz & Schultz, 2008), simulated epiphyte dynamics over 600 years, and compared model results with observations.

Model results were influenced by global model parameters and by the interactions of species with different traits. Global parameters influence all species equally and thus the entire community, while trait parameters influence individual species. For all global parameters and all specific‐specific traits, we first determined initial parameter values or ranges based on literature, our expert knowledge or the datasets from Panama and Ecuador (Appendix S3: Table S1). On this basis, species sets were generated by randomly selecting traits from the trait ranges considering trait correlations. Simulations were carried out, and the effects of uncertain parameters on model results were tested. We observed that epiphyte dynamics were particularly sensitive to four parameters, of which two were global parameters (intercept *k*_M_ of body mass‐dependent mortality probabilities; scaling factor *g*_S_ relating epiphyte biomass to occupied area), one was a trait (average potential number of recruits under optimal conditions *n*_RPot_), and one a global parameter defining trait correlations (intercept *k*_Mat_ of the relationship between the age at maturity and the maximum mass of a species). We varied each parameter five times (respectively, the trait range in the case of *n*_RPot_), which resulted in 625 parameter combinations (25 combinations of global parameters with 25 species sets generated based on different trait ranges and trait correlations). However, none of the 625 parameter combinations resulted in communities in long‐term dynamic equilibrium, that is, the communities were either declining or increasing in abundance. We therefore identified the ten parameter combinations that contained the largest number of species with average population growth rates between 1.00 and 1.01 over the first century, corresponding to species that survive and coexist without quickly outcompeting one another. For each of the ten parameter combinations, we repeated the described procedure (random generation of species set, long‐term simulations, identification of suitable species) until at least 1,000 viable species were identified, from which ten species sets (from now on treated as replicates) with 100 species each were generated. Simulation runs over 600 years were carried out, and the best parameter combination was identified by contrasting the resulting dynamics and structural patterns to the observations (see Appendix S3: Table S1 for parameter values and trait ranges after calibration). The best parameter combination was assumed to be the calibrated model, and its 10 replicate species sets were used in the experiments explained in the next section.

### 2.3 Simulation experiments

After calibrating the model, we carried out three explorative simulation experiments to assess how forest dynamics affect the dynamics of epiphyte communities. For this purpose, the ten species sets of the calibrated model were simulated in different forest systems. For each forest system, five forest replicates were simulated and used as input data for epiphyte simulations, which resulted in 50 simulations per forest system (5 forest replicates × 10 epiphyte species sets). In all experiments, the 100 species of the species sets were initialized in identical numbers at a density of 40,000 individuals per hectare in forests in equilibrium and simulated for 600 years.

In the first simulation experiment, we assessed the effect of different natural forest dynamics on the dynamics of the epiphyte communities. We generated three forest scenarios differing in stem turnover rates in addition to the reference forest. These scenarios are referred to as high‐turnover, low‐turnover, and very‐low‐turnover scenario (Appendix S3: Figure S1). In all scenarios, the turnover rates show annual variation (Appendix S3: Figure S1), with the average annual rates being ~1.6% (very‐low‐turnover scenario), ~2.2% (low‐turnover scenarios), ~2.7% (reference forest), and ~3.2% (high‐turnover scenario). These stem turnover rates represented variations observed in tropical rainforest (between 1% and 4% per year; e.g., Lewis et al., 2004; Phillips, 1996; Phillips et al., 2004). Please note that due to the complex interaction in forests, such variations in turnover rates were also linked to variations in other attributes such as the total basal area or the number of stems. Additionally, we generated a scenario in which the reference forest was static (without any dynamics).

In the second simulation experiment, we assessed the effect of selective logging on the dynamics of the epiphyte communities based on three different logging scenarios differing in the target diameter. These scenarios are referred to as logging40, logging45, and logging50 according to the target diameter at breast height (in cm) for logging (Appendix S3: Figure S2). In each year, all trees reaching the target diameter were removed. This simple management scenario was chosen to assess the importance of larger trees for the epiphyte community. It should be regarded as theoretical scenario, as management decisions are more complex in reality.

In the third simulation experiment, we assessed the effect of fragment size on the dynamics of the epiphyte communities and simulated epiphyte dynamics in the reference forest at three plot sizes (0.25, 0.5 and 1 ha plots; Appendix S3: Figure S3). In these as in the above‐mentioned forest scenarios, forests are isolated and closed systems (fragments) that do not receive any seeds from outside. The light intensity increases toward the forest edge, but other typical edge effects, such as higher tree mortality at the edges, are not represented by the simulated forest input data.

We quantified the effects of forest dynamics on saturation level, abundance, and species richness of the epiphyte communities in each year. Saturation level describes the percentage of arboreal substrate occupied by epiphytes and is hence independent of differences in forest structure (e.g., available surface area). Please note that we used a voxel‐based approach in which individuals were removed from a voxel if their total space requirement exceeded a voxel's available surface area (space competition), meaning that the surface in a single voxel is normally not completely filled. For these reasons, saturation levels of <100% can represent already saturated communities.

**FIGURE 2:**
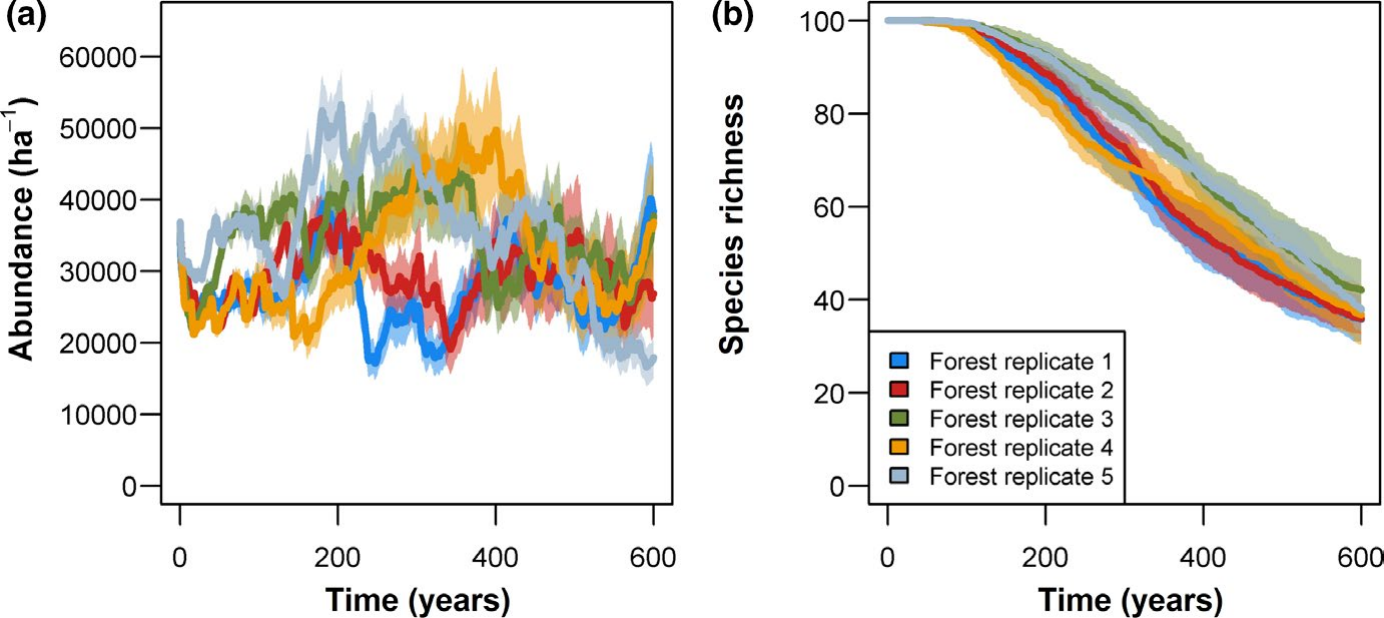
Simulated long‐term dynamics of vascular epiphyte communities. Five replicates of a typical Neotropical lowland forest stand (see Appendix S3: Figure S1 for forest attributes) were used as input data for the epiphyte model. On each of these forest replicates, the development of epiphyte communities, which initially consisted of 100 individuals of 100 species, was simulated over 600 years. Ten different initial species sets were simulated on each forest replicate and means (bold lines) and standard deviations (shaded areas) of abundance (a) and species richness (b) are shown

The model runtime scaled with epiphyte abundance (see Figure 2) and was in the range between 20–60 min for a 600‐year simulation run on an Intel i7 Quadcore (4th generation) using MATLAB (2016).

## 3 RESULTS

### 3.1 Model calibration and validation

Long‐term simulations showed pronounced fluctuations in abundance that varied between 20,000 and 60,000 individuals ha^−1^, indicating a highly dynamic equilibrium state (see Figure 2 for dynamics averaged per forest replicate and Appendix S3: Figure S4 for community dynamics of all simulation runs). These fluctuations were more strongly influenced by differences in forest dynamics among forest replicates than by differences in species sets (Appendix S3: Figure S4). Annual community growth rates ranged from ~0.9 to ~1.05 a^−1^ (see example in Appendix S3: Figure S5a). Drastic reductions in abundance due to tree fall were compensated by positive community growth in periods without substantial tree deaths. Overall, community‐wide annual mortality rates averaged over all replicates were ~14.2% a^−1^ (Appendix S3: Table S2). On average, ~3.5% a^−1^ of all individuals fell to the ground attached to branches or trunks, and ~3.4% a^−1^ died due to competition. Mortality due to changing environmental conditions following changes in forest structure was less important with 0.5% a^−1^. The mass‐dependent mortality rate (which includes seedling mortality) was ~6.7% a^−1^. All species survived the initial ~80–100 years, but subsequently an increasing number of species went locally extinct (Figure 2b).

**FIGURE 3:**
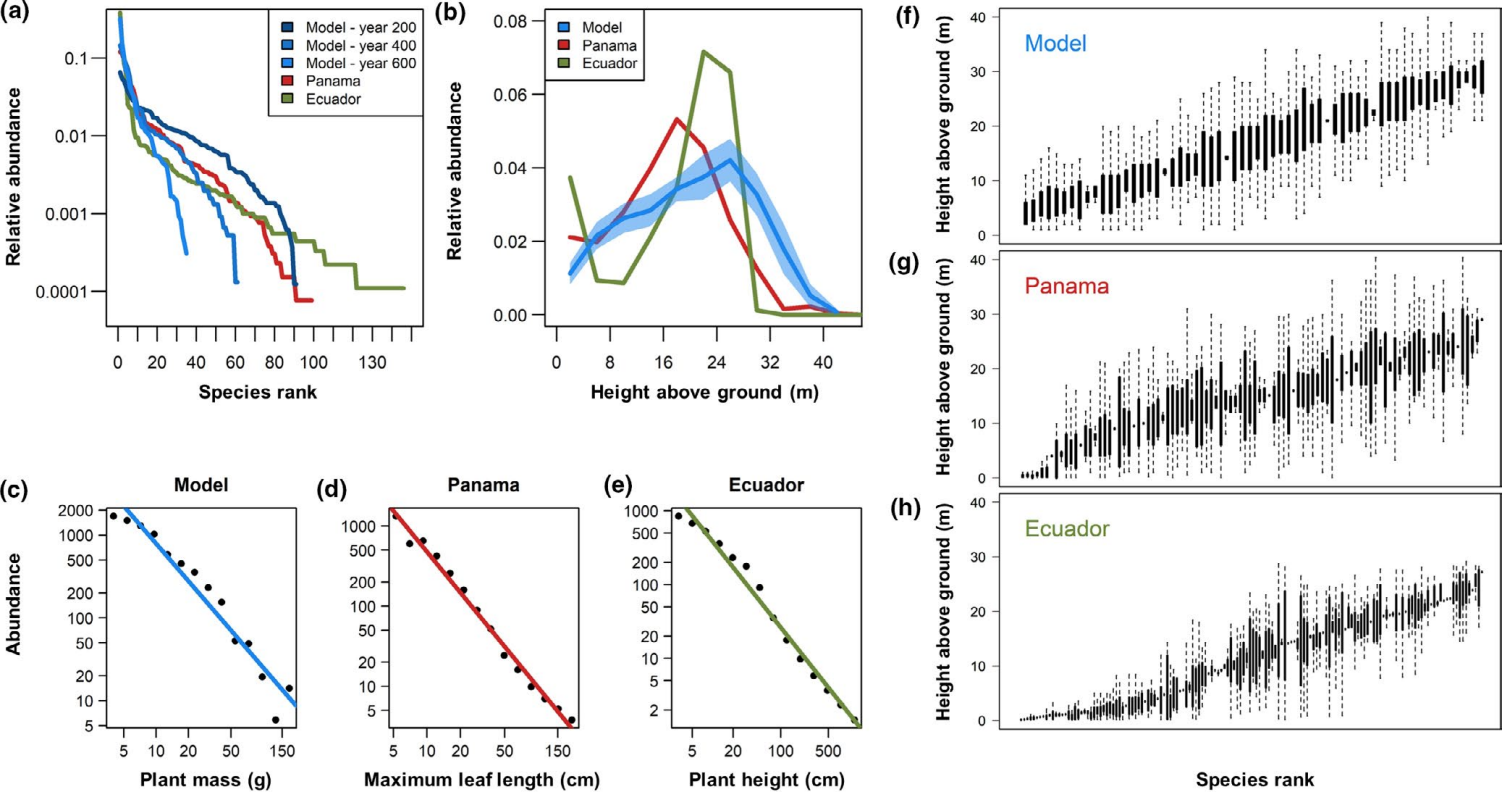
Rank abundance distributions and vertical distributions (a‐b), size‐distributions (c‐e), and vertical stratifications (f‐h) of simulated epiphyte communities in comparison with data from Panama and Ecuador. (a) Relative abundances of species sorted by their abundance rank in descending order in one representative model run at several time steps in comparison with empirical data from rainforests in Panama and Ecuador. (b) Simulated vertical distribution of epiphytes in comparison with empirical data from Panama and Ecuador. The simulated vertical distribution ± standard deviation was calculated based on the pooled model results of the years 300–600 in one representative model run and resulted from an upward shift in abundances from the initial distribution (see Appendix S3: Figure S6). (c‐e) Simulated size distribution was calculated based on the pooled model results of the years 300–600 in one representative model run. Please note that due to limited availability, different proxies for plant size are plotted. (f‐h) Vertical stratification is represented as height distribution for each species, arranged by mean height. The simulated stratification after 300 years is shown for one representative model run

At dynamic equilibrium state, rank abundance distributions were right‐skewed. However, rare species (e.g., singletons) were comparatively underrepresented (Figure 3a). Epiphytes were not evenly distributed vertically in the canopy. Rather, relative abundance peaked at c. 25 m, that is, c. 60% of canopy height (Figure 3b). Furthermore, the simulated communities were size‐structured and dominated by smaller individuals (Figure 3c‐e). Overall, the realized vertical stratification of species resembled empirical patterns of the reference communities, but species with narrow height niches occurring near the forest floor (i.e., “lower trunk specialists”) were not well represented in the model (Figure 3f‐h).

### 3.2 Simulation experiments

**FIGURE 4:**
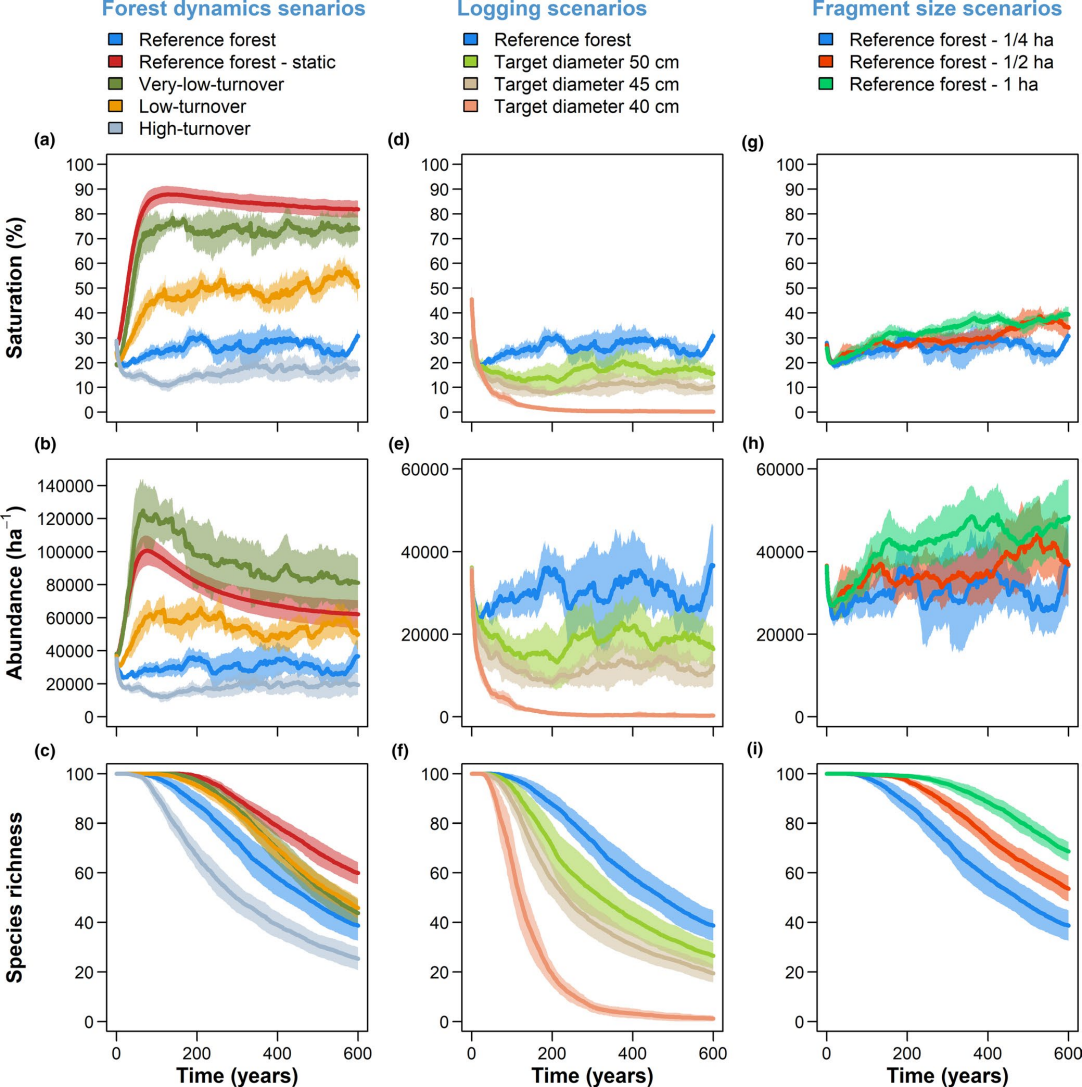
Saturation level, abundance, and species richness of simulated epiphyte communities in forests differing in dynamics, logging regimes, and fragment size. Each panel shows the averaged temporal development of epiphyte communities over 600 years: (a‐c) Forests differing in their natural dynamics (Appendix S3: Figure S1), (d‐f) forests differing in their logging intensity (Appendix S3: Figure S2), and (g‐i) forests differing in their fragment size (Appendix S3: Figure S3). For each of these forest scenarios, five replicates were simulated and used as input data for the epiphyte model. Ten different species sets of vascular epiphytes were separately simulated for each forest replicate. Thus, for each forest scenarios, a total of 50 epiphyte simulations were conducted, and mean values (bold lines) and standard deviations (shaded areas) are shown here. Note that the communities in the first 100–200 years are still re‐assembling from the evenly distributed initial conditions (100 individuals per species) due to typically slow dynamics. Therefore, differences among scenarios are more apparent after 100–200 years

Forest dynamics influenced the saturation level, abundance, and richness of epiphyte communities (Figure 4a‐c). Saturation reached relatively stable levels that were distinguishable among forest scenarios (Figure 4a). In the static scenario (nondynamic forest), epiphyte communities occupied ~85% of total available substrate area. This saturation level was almost reached in the forest scenario with very low tree turnover rates, but all other scenarios were below this level, with decreasing saturation levels with increasing turnover rates (Figure 4a). Epiphyte abundance followed similar patterns for the nonsaturated forests, but decreased over time in the almost saturated forests (static, very‐low‐turnover; Figure 4b). Abundance was highest in the very‐low‐turnover scenario. The number of species surviving until the end of the simulation also differed among scenarios, with increased stem turnover leading to fewer species (Figure 4c). The low‐ and very‐low‐turnover scenarios yielded almost identical species richness, but abundance and saturation levels differed more evidently. Epiphyte communities in selectively logged forests had generally lower saturation levels, abundances, and species numbers compared to unlogged forests (Figure 4d‐f). A reduction in target diameter for logging from 45 to 40 cm resulted in a near‐complete extirpation of the epiphyte community. While fragment size had little effect on saturation level and abundance (Figure 4g‐h), it did affect species richness, with increasing species numbers with increasing fragment sizes (Figure 4i).

## 4 DISCUSSION

### 4.1 Model calibration and validation

Epiphyte abundance fluctuated substantially between the different replicate runs but was relatively stable when averaged over replicate runs (compare Figure 2 and Appendix S3: Figure S4 with the reference forest scenario in Figure 4b). Direct comparisons with observations are currently not possible as there is no information on long‐term community dynamics of epiphytes. However, studies covering up to 7 years invariably reported an increase in abundance (Einzmann & Zotz, 2017; Laube & Zotz, 2006; Schmit‐Neuerburg, 2002), suggesting that epiphyte communities are typically not saturated. Our model results are in line with these observations as we obtained similar positive trends, which were punctuated by tree fall events that prevented the epiphyte community from reaching saturation (Appendix S3: Figures S4 and S5). Hence, our results indicate that gap dynamics provide a mechanistic explanation for unsaturated epiphyte communities. This is particularly true when large trees are involved. For instance, Zotz and Schultz (2008) reported that a single large tree hosted almost 15% of all epiphytes in their 0.4 ha plot. Therefore, large fluctuations in total abundance, controlled by forest dynamics, characterize local epiphyte communities.

Mortality rates were also experiencing substantial annual fluctuations (Appendix S3: Figure S5 and Table S2). The simulated average mortality rate of ~14% a^−1^ agrees well with values reported by Hietz (1997) for a montane forest in Mexico (~16% a^−1^). Mortality rates usually decline with plant size (Zotz & Schmidt, 2006), linked to an increasing resilience to drought (Winkler et al., 2005; Zotz et al., 2005). This frequently observed size‐structured mortality pattern was adequately captured by the implemented metabolic constraints in the model. Additional causes of mortality may result from epiphyte–forest and epiphyte–epiphyte interactions. Mortality rates due to competition (3.4% a^−1^) and due to branch fall (3.5% a^−1^) were in the same order. In comparison with the ranges reported in the literature (4% in Sarmento Cabral et al., 2015, although not considering tree fall; 7% in Hietz, 1997), simulated mortality due to branch fall was slight underestimated, whereas observations in forests with low epiphyte densities suggest that mortality rates due to competition were overestimated in the simulations (Zotz & Vollrath, 2003). However, in our model we also counted individuals whose space requirement exceeded the available space as having died from (space) competition. In reality, such individuals would likely fall from the branch and hence be counted as mortality due to branch fall. This might hence explain the deviations from observations. Overall, the various representations of mortality in our model seem plausible.

Contiguous old‐growth forests are usually characterized by rather stable or even increasing species numbers (Benavides et al., 2006). In contrast, species numbers generally declined over time in our model, which is arguably related to our experimental design, which assumed a closed system without immigration. Consequently, increasing species numbers were not possible in our simulations and local extinctions could not be compensated by recolonization. Our simulations were initialized with 100 species in identical numbers (100 individuals in a 50 × 50 m forest fragment), and due to this setup, there was a lag phase of 100–150 years before the first species became locally extinct. Mortality due to competition and branch fall were important drivers for these local extinctions (Appendix S3: Table S2 and Figure S5). Particularly high mortality rates >20% a^−1^ were observed in years when big trees fell. Such scattered years with high mortality rates were particularly threatening for populations of vulnerable species with already low numbers of individual or restricted to a single or few trees. While the simulated temporal dynamics of species numbers are not comparable to observations in real‐world forests (open systems), they allow comparing the different simulation experiments.

In contrast to our limited understanding of community dynamics, epiphyte community structure is relatively well studied. Rank abundance distributions are usually right‐skewed with only few highly abundant and several rare species (Benavides et al., 2005, 2011; Janzen et al., 2020; Laube & Zotz, 2006). Our model was able to reproduce this right‐skewed rank abundance distribution but underestimated the relative proportion of very rare species (Figure 3a). Again, this might be due to simulating a closed system. As such, rare species inevitably go extinct. The lack of rare species therefore likely results from the lack of metacommunity dynamics in our experiments, which is central to maintain locally rare species populations in patchy geographic distribution in many real‐world landscapes (Janzen et al., 2020). In addition, we observed that smaller species went locally extinct more often (Appendix S3: Tables S3–S4). The mass‐dependent competition in our model (large individuals outcompete smaller ones when space is limiting) might therefore contribute to decrease persistence of rare small species over a longer period.

A pronounced vertical stratification, which is a key feature of real epiphyte communities (Krömer et al., 2007; Petter et al., 2016; Zotz, 2007), also emerged in our experiments, but with less pronounced variation in height ranges (Figure 3f‐h). The applied light niche‐based approach should be appropriate to approximate potential niches of many species, but species with more stringent and complex environmental requirements, for instance trunk specialists such as Hymenophyllaceae (Krömer et al., 2007), may require further model development to capture additional niche axes (e.g., air humidity, temperature, bark texture; Zotz, 2016), which we explicitly neglected here for simplicity.

Epiphyte abundance peaked at intermediate height, which is consistent with the reference epiphyte communities (Figure 3b; Appendix S3: Figure S6). These high abundances coincide with abundance peaks in the inner crowns of large trees (Johansson, 1974; Woods et al., 2015), which have been attributed to a favorable microclimate (Benzing, 1990; ter Steege & Cornelissen, 1989). However, Zotz and Schultz (2008) speculated that such pattern might, at least partly, reflect differences in available substrate area. The central role of substrate area in our model thus strengthens this speculation.

In summary, both composition and structure of the epiphyte communities were adequately emulated by our model. This supports the usefulness of our model for investigating the link between forest structure and dynamics and structurally dependent epiphyte communities.

### 4.2 Simulation experiments

Epiphyte abundance and saturation levels in natural systems vary greatly between regions and tend to peak under mild, frost‐free, humid climates (Küper et al., 2004). Environmental differences, however, also affect forest structure (Asner et al., 2013; Girardin et al., 2010, 2013) and dynamics (Galbraith et al., 2013; Stephenson & Van Mantgem, 2005), which in turn influence epiphytes. In our simulation experiments, the direct effect of forest dynamics on the epiphyte community was large, with the saturation levels in dynamic equilibrium ordered according to turnover rates of the forests (Figure 4a). This finding suggests that forest dynamics are crucially controlling saturation, abundance, and richness of epiphyte communities.

Disentangling the relative effects of the various ecological drivers in epiphyte habitats is not straightforward. Ding et al. (2016) investigated the importance of abiotic and biotic factors along an elevation gradient and found that humidity was of similar importance as forest structure (i.e., basal area) in explaining epiphyte abundance and diversity. These authors speculated that the significant effect of forest structure might be due to an increase in surface area or time for colonization. We explicitly simulated different forest dynamics scenarios, with some properties of dynamics and structure being correlated, as is common in natural forests (Appendix S3: Figure S1; Vilanova et al., 2018). Forests with low‐turnover rates also had a higher basal area and biomass, but the increasing epiphyte abundance in these forests could not be explained solely by the increase in available substrate area, as saturation levels also differed among forests. In other words, the high epiphyte abundance in the low‐turnover scenarios was due to greater substrate availability *and* due to the fact that that the substrate was more densely populated. This indicates that time for colonization, that is, the average turnover rate of epiphyte substrate, has a decisive influence on establishment and mortality and thus on the structure and composition of epiphyte communities.

In general, positive correlations between abundance and species richness are reported for epiphytes (Ding et al., 2016; Zotz, 2016). Our simulations revealed similar correlations between abundance, saturation level, and richness, with noteworthy deviations. For instance, the generally higher abundance despite lower saturation level in the very‐low‐turnover scenario, compared to the static reference scenario, can be explained by the larger arboreal surface area due to a higher density of large trees (Appendix S3: Figure S1). In these two scenarios, the decrease of abundance with time after c. 100 years at relatively stable saturation levels is the result of the simulated mass‐dependent asymmetric competition where larger individuals outcompete smaller ones (see Appendix S3: Table S3–S4). Increased mortality rates due to competition were also the main reason for the decline in species richness in the static reference scenario after c. 200 years. In the very‐low‐turnover scenario, saturation and mortality due to competition were at similar levels as in the static scenario, but mortality due to branch fall was higher (no such mortality in the static forest; Appendix S3: Figure S8), which resulted in overall higher rates of local extinction. In general, mortality due to branch and tree fall increased from the static to the high‐turnover scenarios, while mortality due to competition decreased (Appendix S3: Figure S8), and this provides an explanation why the richness was similar in the low‐ and very‐low‐turnover scenario. In fact, the risk of local extinction increases with increasing forest turnover rates, particularly for slow‐growing species, but also with increasing saturation level, particularly for species with low competitiveness. While it is still debated whether competitive exclusion plays an important role in epiphyte communities (Benavides et al., 2005; Flores‐Palacios & Garcia‐Franco, 2006; Zotz et al., 1999), our results indicate that competition can be more relevant in forest systems with low forest turnover rates, for example, in montane forests (Stephenson & Van Mantgem, 2005).

In our second experiment, the reduction of the target diameter for logging to 40 cm had a catastrophic effect on epiphyte abundance and richness (Figure 4d‐f). In this scenario, the above‐ground biomass and the biomass residence time were lowest, that is, the surface area for epiphytes was the smallest and available for the shortest time (Appendix S3: Figure S2). Under these conditions, only very few, predominantly small species with higher growth rates managed to survive at low abundances (Appendix S3: Table S3). This suggests that particularly susceptible species with slow demographic rates require stable habitats provided by large, old trees to reach maturity and to maintain viable populations. For the specific species set in our simulations, a target diameter of 40 cm was an important threshold. However, this must be interpreted with caution and is not directly transferable to other epiphyte communities in the real world. Such thresholds could exist, but they would strongly depend on the local forest dynamics and the characteristics of members of the local epiphyte community.

Negative effects of logging on epiphyte communities were also observed by Padmawathe et al. (2004). They sampled natural and logged forest years after logging had stopped, and still observed negative effects of logging on abundance and diversity for some, but not all, taxonomic epiphyte groups. Our results thus support the particular importance of large trees for epiphyte conservation. Large, old trees often host a large number of epiphyte individuals and species because they provide high microhabitat heterogeneity (Grubb et al., 1963; Hundera et al., 2013; Woods, 2017; Zotz & Schultz, 2008) and relatively stable habitat (their trunk and inner crown are available over decades to centuries), allowing more time for species accumulation (Taylor & Burns, 2015; Wagner & Zotz, 2020). Additionally, the stable habitats also promote the accumulation of canopy humus in the inner crowns in montane forests (not simulated here), which can further increase abundance and diversity of epiphytes (Woods et al., 2015). Therefore, biodiversity‐friendly forest management must include sparing some large trees to safeguard epiphyte diversity (Jones et al., 2018; Lindenmayer et al., 2012).

In the third simulation experiment, the rate of local species loss increased with decreasing fragment size, that is, in larger fragments a more diverse epiphyte community was maintained in the long run (Figure 4i). This result agrees with many observations of lower species richness of many organisms in smaller forest fragments (Martensen et al., 2008; Pardini et al., 2005). Edge effects or limited immigration are often discussed as probable reason for this pattern (Turner, 1996). These effects, however, do not play a role in our model when comparing the different scenarios. Here, the disproportionate effect of local disturbances caused by gap‐creating tree fall events in smaller fragments increases local extinctions. In real‐world systems, the impact of fragmentation should be even worse because epiphytes are also affected by drier microclimates at the forest edges (Cascante‐Marín et al., 2009; Flores‐Palacios & García‐Franco, 2007), which was not simulated. In line with previous studies, our results thus emphasize the importance of large tracts of intact forests for epiphyte conservation (Flores‐Palacios & García‐Franco, 2007; Hundera et al., 2013).

### 4.3 Limitations and outlook

Every model has limitations. The presented model was developed to learn more about possible effects of forest dynamics on epiphyte dynamics. For this purpose, virtual epiphyte species were generated, and the dynamics of epiphyte communities were simulated in several simulation experiments that used 3D forest dynamics simulations as input data. This modeling setup allowed simulating and evaluating an identical set of epiphyte species on different forest systems ‐ a comparable experimental setup is hardly imaginable in reality. By using this modeling setup, we observed, for instance, that differences of just 1% in forest turnover rates could have a substantial impact on the abundance and saturation level of epiphyte communities, or that the reduction of the target diameter for logging could have drastic consequences for the epiphyte community. These results indicate the importance of forest dynamics for epiphyte communities but should be interpreted with caution: They are not meant to be used as quantitative predictions.

It is also important to note that in its current state, our model is not suitable as management tool. This is mainly due to the fact that lacking data on epiphytes hampers model parameterization and validation. However, precisely because of these data limitations, modeling approaches are important tools, as they allow exploring hypotheses in a theoretical framework. Moreover, gathering empirical data can actually profit from models such as ours as these i) present the parameters that can be calibrated with empirical data and ii) provide predictions to be tested as hypotheses by field studies. Further model development can then be informed by these model‐inspired empirical studies. Hence, model development is a long process that takes place in interaction with empirical data in the so‐called modeling cycle (see Hartig et al., 2012 for forest modeling).

Our trait‐based model considers interspecific differences based on biomass according to the metabolic theory of ecology, direct influences of forests on establishment and mortality of epiphytes, and the influence of within‐canopy light conditions on growth. However, ecophysiological responses to other environmental factors such as humidity or nutrient availability are not yet simulated explicitly. To consider ecophysiological interactions mechanistically in future model developments, improved knowledge of how the community composition is influenced by environmental conditions is needed, as well as quantitative ecophysiological knowledge at the level of species or functional groups. Such information is still very scarce (Zotz, 2016). Other extensions may include a better representation of different types of reproduction, for example by adding a species‐specific ability of producing few near‐by, half‐grown seedlings to explicit consider clonality. In spite of such limitations, our model proved to be useful to qualitatively explore the effects of different forest systems on epiphyte communities.

Another source of uncertainty is related to the forest input data. Although the forest model has been calibrated extensively (Petter et al., 2020), we expect that ongoing development in terrestrial and airborne laser scanning technologies will further improve the calibration of structural forest models. Repeated LiDAR data of canopy substrate could also be directly used as forest input. However, we stress that any model is necessarily a simplification of reality, and one of the main challenges is to determine the right level of complexity. Increasing the level of complexity, for instance by implementing further alternative functions for growth or reproduction, adds model uncertainty (Jeltsch et al., 2008). Explicitly considering model uncertainty or increasing mechanistic complexity should match the purpose of the model, taking into account whether there is adequate empirical data available for model selection (Cabral & Schurr, 2010). It is thus common to start with a simple version of the model (see review in Cabral et al., 2017) and verify how much it already represents the system in general terms. For a model to explain a system in detail, follow‐up development based on detailed data is generally necessary for a successful modeling cycle.

Whereas some of the above‐mentioned limitations can be addressed in future research, mechanistic modeling studies as presented here already improve our understanding of epiphyte ecology (also see Spruch et al., 2019): They disentangle cause and effect in the highly dynamic and structurally complex epiphytic environment and they cover intervals which are relevant for epiphyte dynamics, but that escape the time span of field studies. Future model studies could design simulation experiments to address metacommunity dynamics by connecting several forest patches via seed dispersal or analyze trait composition and structure in dependence of forest dynamics in detail. There are also perspectives for conservation: In the future, extended versions of our model and of our experimental design might be used to analyze the impact of more realistic logging schemes or to inform mitigation possibilities to impede local extinctions after human‐induced perturbations (see Figueiredo et al., 2019 for a review).

## 5 CONCLUSIONS

By developing the first niche‐based, demographic model for epiphytes, our framework promotes explorative modeling for a species‐rich life‐form that has been largely neglected in mechanistic modeling and environmental change assessments. Our model demonstrates that the abundance and diversity of epiphyte communities is tightly linked to forest dynamics, structure, management, and forest fragment size. Our results suggest that expected future changes in tropical forests, such as increased turnover and mortality rates, will have a negative effect on the structure and dynamics of epiphyte communities. Additional processes related to direct human pressure, such as logging and fragmentation, pose even more challenges to epiphyte conservation. All these findings provide cautionary lessons and stress the need for improving our ecological knowledge of epiphytes.

## CONFLICT OF INTEREST

The authors have no conflict of interest to declare.

## AUTHOR CONTRIBUTIONS

**Gunnar Petter:** Conceptualization (supporting); Formal analysis (lead); Methodology (lead); Software (lead); Validation (lead); Visualization (lead); Writing‐original draft (lead); Writing‐review & editing (lead). **Gerhard Zotz:** Conceptualization (supporting); Supervision (supporting); Writing‐original draft (supporting); Writing‐review & editing (supporting). **Holger Kreft:** Conceptualization (supporting); Supervision (lead); Writing‐original draft (supporting); Writing‐review & editing (supporting). **Juliano Sarmento Cabral:** Conceptualization (lead); Methodology (supporting); Supervision (lead); Validation (supporting); Writing‐original draft (supporting); Writing‐review & editing (supporting).

## Supporting information

Appendix S1

Appendix S2

Appendix S3

## Data Availability

The model source code is available at GitHub (https://github.com/julianoscabral/MoDVE). Validation data have been deposited in Dryad (https://doi.org/10.5061/dryad.cvdncjt3h).
