# Supplementary material for: Agent‐based modeling of the effects of forest dynamics, selective logging, and fragment size on epiphyte communities
Source: Ecol Evol. 2021 Feb 28;11(6):2937–51. doi: 10.1002/ece3.7255 (PMC7981202; doi:10.1002/ece3.7255)
Supplement: Supplementary file 1 — Appendix S1 [file ECE3-11-2937-s001.pdf]

Appendix A1

Emergent results of the calibrated forest model used to generate input data for the epiphyte model

to

Agent-based modeling of the effects of forest dynamics, selective logging, and fragment size on epiphyte communities

Gunnar Petter, Gerhard Zotz, Holger Kreft, Juliano Sarmento Cabral

In this appendix, we show key emergent results of the 3D functional-structural forest model calibrated for Neotropical lowland forests (Petter et al., 2020), which was used to generate input data for the epiphyte model. In the forest model, trees are represented as dynamically growing, functional-structural tree models. Branches up to the second order are considered and leaf biomass dynamics are modelled at a resolution of 1 m^3, allowing simulations of competition for space and light at a high level of detail. Trees are characterized by functional traits, and in this way different tree types from fast-growing short-living to slow-growing long-living ones are represented in the model. A visualization of a simulated forest plot at different years is provided in Fig. S1. Forest attributes in comparison to typical ranges of these attributes in Neotropical forests are shown in Fig. S2. Additional patterns of the simulated forest are shown in Fig. S3.

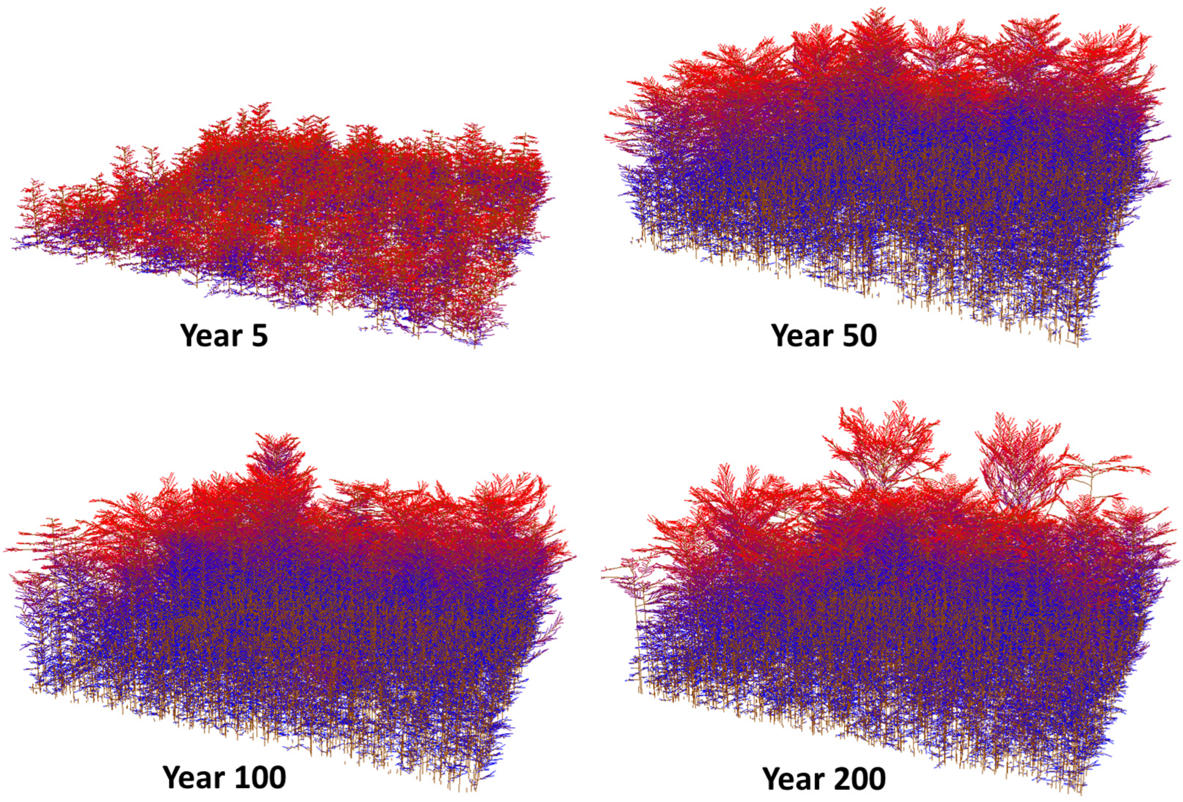

Fig. S1. Visualizations of the simulated forest plot at different years. The simulation started from bare ground and was initialized by randomly dispersing seeds from a species set containing 1000 tree species differing in their functional and structural traits. In this visualization, branches of the trees are colored according to the light conditions (high light intensities: red, low light intensities: blue). Please note that leaves are not shown here, and that trunks and branches are represented by cylinders of the same diameter, i.e. not to scale.

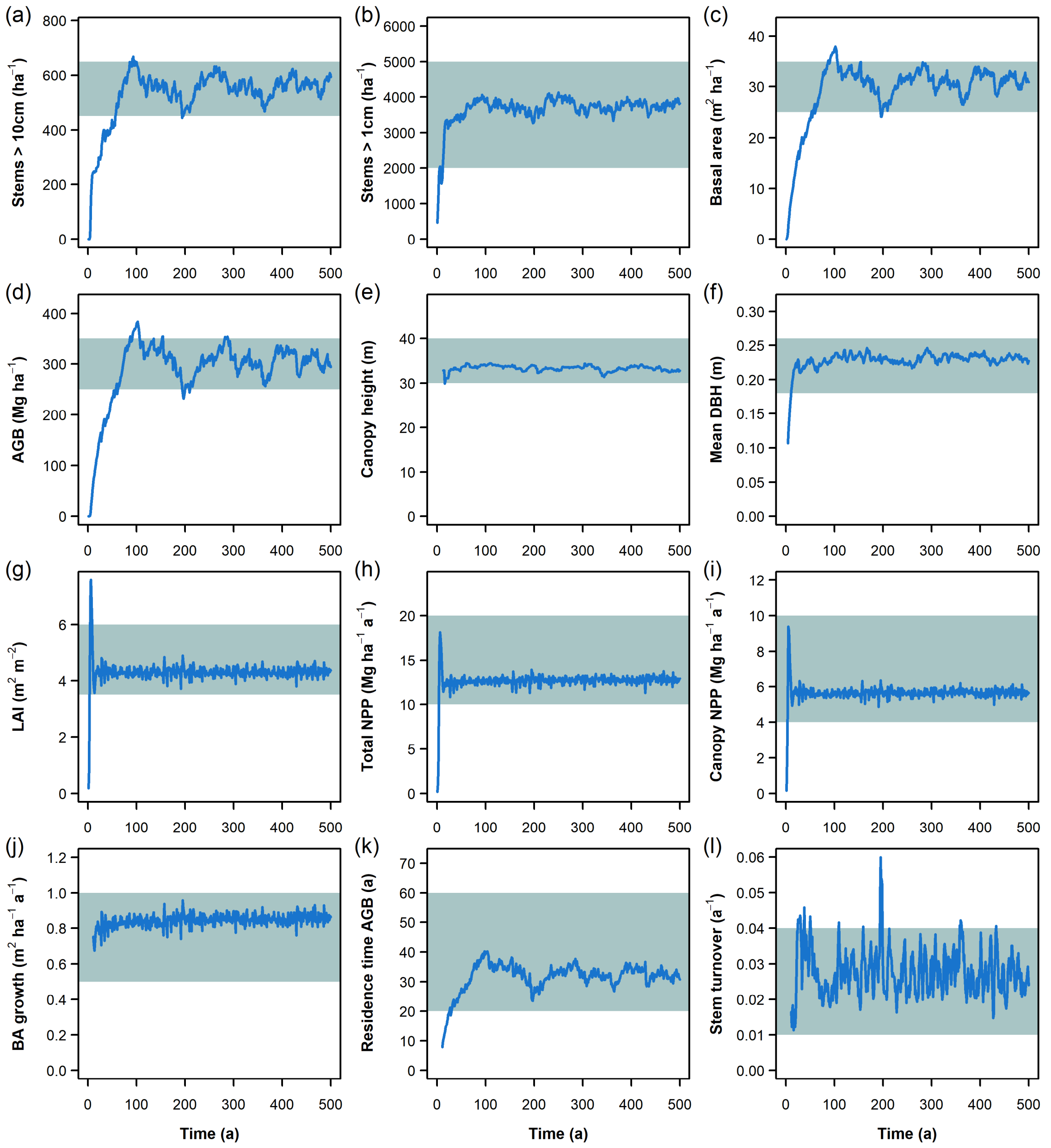

Fig. S2. Simulated long-term dynamics. A 50x50 m forest plot was simulated with a functional-structural forest model over 500 years and the following forest attributes were recorded: (a) Stem density of all stems with diameter at breast height (dbh) > 10 cm, (b) Stem density of all stems with dbh > 1 cm, (c) Basal area, (d) Above-ground biomass (ABG), (e) Canopy height (mean height of all trees > 40 cm in dbh), (f) Mean dbh of all stems > 10 cm in dbh, (g) Leaf area index (LAI), (h) Total above-ground net primary production (NPP), (i) Canopy net primary production (NPP of leaves and second order branches), (j) Basal area (BA) growth, (k) Residence time of above-ground biomass, (l) Turnover of all stems > 10 cm in dbh. Typical ranges of attributes of Neotropical forests were estimated based on a literature review and are indicated here by the grey-shaded areas.

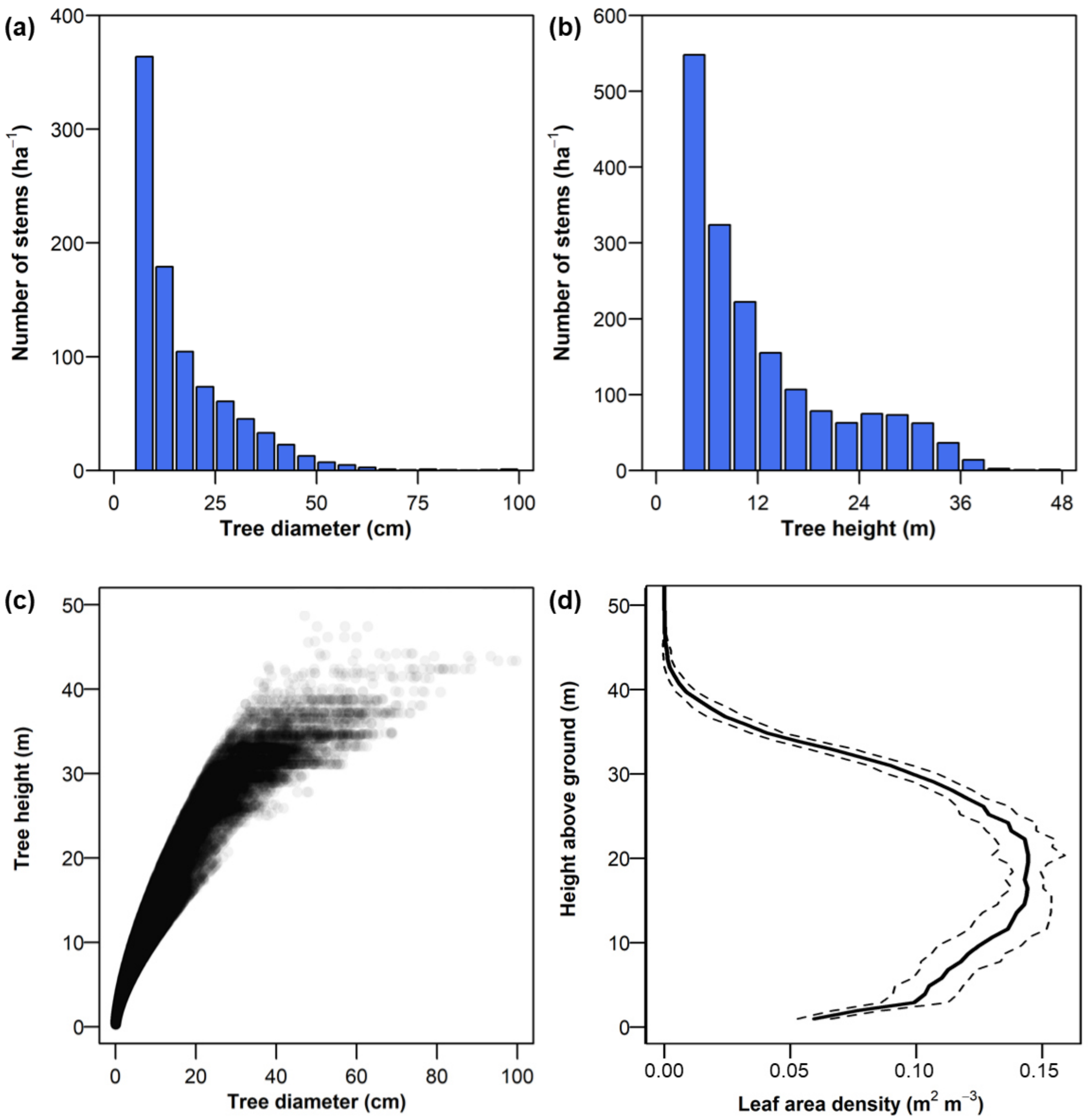

Fig. S3. Frequency distribution of diameter at breast height (a) and tree height (b), height-diameter relationship (c), and vertical leaf area density distribution (d). The frequency distributions and the leaf area density distribution show means over the years 200-500 (forest in dynamic equilibrium). To reduce the degree of temporal pseudoreplication, trees considered for the height-diameter relationship were sampled in time intervals of 50 years in dynamic equilibrium.

Reference

Petter, G., Kreft, H., Ong, Y., Zotz, G., & Sarmento Cabral, J. (2020). Modeling the long-term dynamics of tropical forests: from leaf traits to whole-tree growth patterns. *bioRxiv*. doi:10.1101/2020.06.01.128256
