# Supplementary material for: Agent‐based modeling of the effects of forest dynamics, selective logging, and fragment size on epiphyte communities
Source: Ecol Evol. 2021 Feb 28;11(6):2937–51. doi: 10.1002/ece3.7255 (PMC7981202; doi:10.1002/ece3.7255)
Supplement: Supplementary file 2 — Appendix S2 [file ECE3-11-2937-s002.pdf]

Appendix A2

Model description

to

Agent-based modeling of the effects of forest dynamics, selective logging, and fragment size on epiphyte communities

Gunnar Petter, Gerhard Zotz, Holger Kreft, Juliano Sarmento Cabral

The model description follows the ODD (Overview, Design concepts, Details) protocol which was proposed as standard protocol to communicate agent-based models (Grimm et al., 2006, 2010)

Purpose

The main purpose of this model is to analyze the influence of forest dynamics on the structure and dynamics of vascular epiphyte communities. Vascular epiphytes germinate and grow on trees. Hence, their fate is connected to the dynamics of their host trees, which grow and create new substrate, but also shed branches and ultimately fall and die (Cabral et al., 2015; Taylor & Burns, 2015; Spruch et al., 2019). Driven by differences in the natural environment or by human interventions, forest dynamics can vary substantially (Brown, Gillooly, Allen, Savage, & West, 2004; Quesada et al., 2012; Wright, 2005). We studied the impact of such variations on epiphyte communities.

Entities, state variables and scales

The epiphyte model is three-dimensional and voxel-based, and its spatial extent depends on the spatial dimensions of the input forest data. Here, forests cover an area of 0.25 to 1 hectare and have a canopy height of max. 50 m. The model space is subdivided into voxels of 1 m^3, whose state variables characterize three key environmental conditions: i) light intensity, ii) total area of arboreal substrate, and iii) relative loss of substrate area (Table S1). We acknowledge that there are other abiotic factors varying within a forest. While some of these, such as humidity, vary in concert with light intensity (Wagner, Bogusch, & Zotz, 2013) and are thus included implicitly, others like bark texture (substrate quality) are ignored at this model stage. The model proceeds in annual time steps and the state variables of the voxels are updated each year according to the input data (Fig. S1). Individual epiphytes are the ecological entities whose growth, reproduction, and mortality are simulated as functions of their ecological traits and of the environmental conditions in the voxels. The state variables and traits of epiphytes are summarized in Table S1.

Table S1. State variables and species-specific traits. The demographic processes and the state variables of individual epiphytes are influenced by the state variables of the voxels (i.e. environmental conditions), and by the specific traits of each species to which an individual epiphyte belongs.

Symbol	Description	Unit	Type
A	Age of epiphyte	year	State variable (epiphyte)
E_X, E_Y, E_Z	Position of epiphyte in model space in X, Y, Z direction	m	State variable (epiphyte)
ID_{Ind}	Identifier of epiphyte individuals	-	State variable (epiphyte)
ID_{Sp}	Identifier of epiphyte species	-	State variable (epiphyte)
M	Mass of epiphyte	g	State variable (epiphyte)
I	Light intensity	μmol m^{-2} s^{-1}	State variable (voxel)
S_B	Total surface area of arboreal substrate	m^2	State variable (voxel)
S_{Loss}	Percentage annual surface area change in voxel	%	State variable (voxel)
V_X, V_Y, V_Z	Position of voxel in model space in X, Y, Z direction	m	State variable (voxel)
A_{Mat}	Age at maturity	year	Species-specific trait
D_K	Dispersal ability - factor B in negative exponential function	-	Species-specific trait
D_{KAs}	Dispersal kernel asymmetry	-	Species-specific trait
I_A, I_B, I_C	Parameters A, B, C of parabolic light response curve	-	Species-specific trait
I_{Min}, I_{Max}, I_{Opt}	Minimum, maximum, optimum light intensity for survival	μmol m^{-2} s^{-1}	Species-specific trait
K	Growth rate (von Bertalanffy growth)	a^{-1}	Species-specific trait
M_{Mat}	Mass at maturity	g	Species-specific trait
M_{Max}	Maximum mass	g	Species-specific trait
n_{RPot}	Average potential number of recruits per individual	#	Species-specific trait

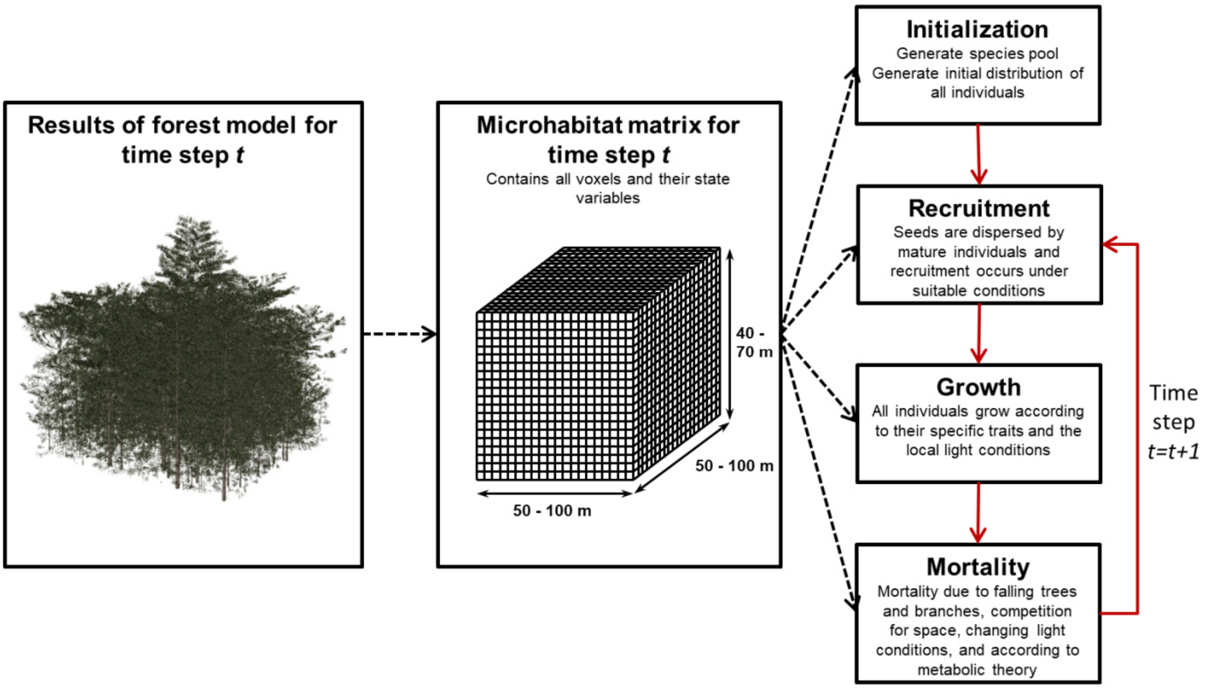

Figure S1. Flowchart of the coupled forest-epiphyte model. Based on dynamic three-dimensional input data from a functional-structural forest model (Petter et al., 2020), a microhabitat matrix characterizing the epiphytic habitat at each time step is generated. To this end, the simulated spatial distribution of leaf area, branches and trunks for each annual time step (left panel) is used to calculate the light distribution, total substrate area and relative annual change of substrate area for each 1 m^3-voxel in the microhabitat matrix (middle panel), which ultimately influences the initialization and all three submodels of the epiphyte model (right panel).

Process overview and scheduling

Based on the forest input data, three-dimensional microhabitat matrices containing the state variables of all voxels are calculated for each annual time step (Fig. S1). The first microhabitat matrix is used to initialize the distribution of epiphytes. After initialization, recruitment, growth, and mortality of each individual are simulated successively at each time step (Fig. S1), as shortly described below.

Recruitment: mature epiphytes reproduce, with the number of new recruits determined by the species-specific fecundity (n_{RPot}, Table S1), the species-specific dispersal kernel (D_K and D_{KAs}, Table S1), and the surrounding substrate area of trees and branches. The actual number of new recruits is calculated based on Poisson random values. The model does not distinguish between sexual and asexual reproduction. However, species with a narrow dispersal kernel, which causes recruits to establish

within the immediate proximity of the mature plant, could also be interpreted as species with mainly clonal reproduction, with each new clone directly being counted as new individual.

Growth: growth of each individual is simulated as a function of its mass and the light conditions in the specific voxel. From the several possible plant growth functions (Paine et al., 2012), we opted for the von Bertalanffy function to achieve generality across species and to maintain a low number of parameters. It describes reasonably well the few known growth trajectories of epiphytes (e.g. Schmidt & Zotz, 2002) and it has only one parameter which can be derived from three traits.

Mortality: individuals die when the light conditions are outside the species-specific light niche, which can occur when the forest structure changes (mortality due to changing environmental conditions). If several individuals occupy the same voxel and their total space requirement exceeds the available surface area, smaller individuals will be outcompeted by larger ones. Mortality also occurs when an individual is alone in a voxel but grows beyond the physical space present in the voxel. The latter two mortality processes summarize resource limitation and competition. Furthermore, individuals may die due to branch or tree fall, with the relative surface loss in a voxel defining the probability of mortality. Additionally, body mass-dependent mortality probabilities following the quarter-power law of the metabolic theory of ecology (MTE; Brown et al., 2004) account for mortality causes not explicitly simulated (e.g. desiccation or pathogens). Mortality probabilities are used to draw individual deaths from a random Binomial distribution.

After this final step, the age of surviving epiphytes is updated and the model proceeds to the next time step.

Initialization

The initial microhabitat matrix describes the light conditions and the distribution of substrate in each voxel and forms the basis of the initial epiphyte distribution. At first, a species set containing traits of a defined number of epiphyte species (here: 100) is generated (for details see *Submodels*). Subsequently, the ratio of juvenile to mature plants in the initial assemblage is specified and, on this basis, a defined number of individuals per species are assigned to each group. Here, we used a total density of 400

individuals per species and hectare. The maximum mass and the mass at maturity are species-specific traits, and the initial mass of each juvenile and adult is randomly chosen from the range [0, mass at maturity] or [mass at maturity, maximum mass], respectively. Subsequently, all individuals of all species are placed in the initial microhabitat matrix. Specifically, this means that for one individual after the other (to avoid biases, the sequence of individuals is randomized), all suitable voxels are estimated and one of these is randomly chosen as initial location. Suitable voxels have light conditions within the species-specific light niche (Table S1) and enough unoccupied surface area for the individual. The initial state of the model thus describes the location, state and species identity of each individual. This initial state is saved, which allows replications using identical initial conditions.

Input

The results of a three-dimensional (3D) functional-structural forest model (FSFM) simulated with the software GroIMP (Growth Grammar Interactive Modelling Platform; available under the GNU General Public License at www.grogra.de) are used as forest input data in this study (Petter et al., 2020). In this FSFM, growth, establishment and mortality of three-dimensional virtual trees is simulated at the stand scale, whereby each tree consist of one trunk and branches up to the 2nd order. Based on the 3D distribution and the dynamics of branches and trunks, the voxel-based distribution of substrate areas and their changes can be estimated. In addition, as the 3D distribution of leaf area is simulated at a resolution of 1m^3, the 3D light environment can also be calculated. These input data thus represent the dynamics of forests at a high level of detail.

Submodels

All symbols and abbreviations used in this section are summarized in Table S2.

Generation of microhabitat matrices

3D microhabitat matrices describing the 3D structure and dynamics of forests are used as input data for the epiphyte model. One microhabitat matrix is required for each time step (here: annual time steps). Each matrix element is called a voxel and represents a cube of $1m^3$ (the size of the voxel is generally not fixed and can be chosen by the model user). For each voxel, the total arboreal surface area, the percentage of surface loss per time step, and the light intensity are essential state variables (Table S1). In this study, this information was generated with a FSFM, but it may also be obtained from other models or observations. In the following, the calculation of the microhabitat matrices on the basis of the FSFM model results is described.

A file containing the start position P_{SStart}^{XYZ} and end position P_{SEnd}^{XYZ} of each branch segment in 3D space, as well its length L_S and diameter D_S, was saved at each time step in the FSFM. Based on L_S and D_S, the surface area of each branch segment S_B is calculated:

$$S_{BS} = \frac{\pi \cdot L_S \cdot D_S}{2} \quad (1)$$

We assume that only the upper branch parts can be colonized by epiphytes, and hence the total surface area of the branch segment is divided by 2.

The maximum internode length used in the FSFM in this study was 50 cm. As the side length of a voxel is 1 m, each branch segment (=internode) may thus only intersect with a maximum of two voxels in X, Y and Z direction. The intersecting voxels in X direction V_{IntX} are calculated as follows:

$$V_{IntX} = unique\left(\text{ceiling}\left(P_{SStart}{}^{X}\right), \text{ceiling}\left(P_{SEnd}{}^{X}\right)\right) \quad (2)$$

Analogously, the intersecting voxels in Y direction V_{IntY} and Z direction V_{IntZ} are estimated. Subsequently, the total number of intersecting voxel can be estimated as:

$$n_{Int} = \text{length}(V_{IntX}) \cdot \text{length}(V_{IntY}) \cdot \text{length}(V_{IntZ}) \quad (3)$$

S_{BS} is evenly split among all intersection voxels and the total surface area in these voxels S_B is updated as:

$$S_B = S_B + \frac{S_{BS}}{n_{Int}} \quad (4)$$

In the vast majority of cases, branch segments are completely contained in a single voxel, or intersect with two voxels, and thus we consider this simplified method as appropriate.

The position of each trunk in X and Y direction P_T^{XY}, as well as its length L_T and diameter D_T, are additional information saved by the FSFM. Trunks are not split into several segments in the FSFM and their total surface areas thus have to be partitioned among intersecting voxel. To this end, we assume that each trunk has the form of a cone and only consider voxels directly above the voxel containing P_T^{XY}. The highest voxel the trunk is intersecting with V_{ZMax} can easily be calculated based on L_T:

$$V_{ZMax} = \text{ceiling}(L_T) \quad (5)$$

For this voxel, the length of the intersecting trunk part L_{TS} is calculated as follows:

$$L_{TS} = L_T - \text{floor}(L_T) \quad (6)$$

As the radius in a cone linearly decreases with height, the radius at the intersection between the trunk part and the voxel r_{Int} can be estimated as:

$$r_{Int} = \frac{L_{TS} \cdot D_S}{L_S \cdot 2} \quad (7)$$

Based on r_{Int} and L_{TS}, the lateral surface of the cone S_T (representing the total trunk surface area in this voxel) can be calculated:

$$S_T = \pi \cdot r_{Int} \cdot \sqrt{{r_{Int}}^2 + {L_{TS}}^2} \quad (8)$$

Analogously, the total lateral surface of the cone spanning from the intersection between the next lower voxel to the maximum tree height can be estimated. To calculate the specific surface area that intersects with this specific voxel only, the trunk surface area from the next higher voxel to the maximum tree height is subtracted. Following this routine from the highest to the lowest voxel, the trunk surface area in each voxel is calculated.

Looping through the trunk and all branch segments following Eqs. 1-8, the total arboreal surface area in each voxel S_V is calculated by summing up all S_T and S_B.

To estimate the relative change of surface area in a voxel S_{Loss} at each time step, the difference in S_V in two consecutive years (*t, t-1*) is calculated and divided by S_V in year t.

The light intensity in each voxel I^{XYZ} is calculated based the total leaf area in each voxel ${A_{LTot}}^{XYZ}$, which was saved at each time step in the FSFM. Based on the sum of ${A_{LTot}}^{XYZ}$ in all voxels above the specified voxel, the leaf area index LAI^{XYZ} for each voxel is calculated.

$$LAI^{XYZ} = \frac{\sum_{Z}^{MaxZ} {A_{Ltot}}^{XYZ}}{{L_V}^2} \quad (9)$$

where L_V is the side length of a voxel. Assuming a Lambert-Beer extinction law, the single-column light intensity ${I_{SC}}^{XYZ}$ is calculated based on LAI^{XYZ}.

$${I_{SC}}^{XYZ} = I_{max} \cdot e^{-(k_L \cdot LAI^{XYZ})} \quad (10)$$

where I_{max} is the light intensity above the canopy and k_L the light extinction coefficient. This method assumes that solar radiation only penetrates directly from above and disregards additional processes like light reflection. This is an oversimplification, particularly in such heterogeneous forests as simulated here. To get a more realistic estimation of the average, effective light intensity within a voxel I^{XYZ}, the single column light intensity $I_{SC}{}^{XYZ}$ in the voxels surrounding the focal voxel in x and y direction are additionally taken into account. The number of surrounding voxels considered depends on the parameter LR which defines how many rectangular rings around the focal voxel are considered. For each considered voxel, the relative contribution C_R is calculated, with $\Sigma\ C_R = 1$. C_R thus defines how strong $I_{SC}{}^{XYZ}$ in each voxel contributes to the effective light intensity I^{XYZ}, and we assume that all rings contribute equally:

$$C_R = \frac{1}{LR+1} \cdot \frac{1}{\max(1, R \cdot 8)} \tag{11}$$

where R is the ring number, counting outwards from 0 (focal voxel) to LR. On this basis, I^{XYZ} is calculated as

$$I^{XYZ} = \sum_{X_{min}}^{X_{max}} \sum_{Y_{min}}^{Y_{max}} I_{SC}{}^{XYZ} \cdot C_R \tag{12}$$

where $X_{min}=X\text{-}LR$ and $X_{max}=X+LR$ (likewise for Y).

Generation of initial species set

At the beginning of each simulation, the local species set containing the trait information of n_{SP} species is initialized. Each species has a unique identifier and is characterized by a set of traits (Table S1). Some traits are independent and randomly chosen from defined ranges, others are related to the body mass following the principles of the metabolic theory of ecology (MTE; Brown et al., 2004).

First, the maximal body mass M_{Max} of each species is specified based on the defined lower and upper limits M_{Max}^{L} and M_{Max}^{U} (the superscripts in this section always refer to the user-defined upper and lower limits of a specific trait). We assume that smaller species are more frequent and thus chose M_{Max} randomly from the uniform distribution after log transformation of M_{Max}^{L} and M_{Max}^{U}:

$$M_{Max} = 10^{\mathrm{unif}(\log({M_{Max}}^{L}), \log({M_{Max}}^{U}))} \quad (13)$$

We assume that the mass at maturity M_{Mat} scales with M_{Max}:

$$M_{Mat} = M_{Max} \cdot \mathrm{unif}({M_{Rel}}^{L}, {M_{Rel}}^{U}) \quad (14)$$

where M_{Rel} defines the ratio between M_{Mat} and M_{Max}. The MTE predicts a positive quarter-power scaling of the age at maturity A_{Mat} with M_{Max} (Brown et al., 2004; Duncan, Forsyth, & Hone, 2007), and accordingly A_{Mat} is calculated as follows:

$$A_{Mat} = \left(k_{Mat} + {M_{Max}}^{1/4}\right) \cdot \mathrm{unif}\left({A_{MatDev}}^{L}, {A_{MatDev}}^{U}\right) \quad (15)$$

where k_{Mat} is the intercept of the A_{Mat}-M_{Max} relationship. To add stochasticity, the relative deviation from the mean trend A_{MatDev} is also considered.

In this model, epiphytes grow according to the von Bertalanffy growth law (see *Growth*), in which the growth rate (or curvature parameter) K is a species-specific parameter. This parameter is calculated based on M_{Max}, M_{Mat} and A_{Mat} as follows:

$$K = -\left(\frac{\log(1) + \log\left(1 - \frac{M_{Mat}}{M_{Max}}\right)}{A_{Mat}}\right) \quad (16)$$

In natural epiphyte communities, a pronounced vertical stratification is usually observed (Krömer, Kessler, & Gradstein, 2007; Petter et al., 2016). The reasons why species occurrences are limited to specific vertical ranges are complex; however, in this model we assume that light defines the niche. In a forest canopy, the light intensity does not vary linearly from the upper strata to the forest floor, but rather non-linearly, commonly described by a light-attenuation law such as Lambert-Beer. Hence, the height niche and the light niche are not linearly related. As vertical niches of epiphyte species are better studied and more intuitive, we at first specify the vertical niche for each species in a standardized forest (Optimum height, minimum height and maximum height relative to the maximum height of the forest), and subsequently translate the vertical niche to the light niche. Thus, at first the relative mean height H_{Mean} is randomly chosen for each species:

$$H_{Mean} = \mathrm{unif}(0,1) \tag{17}$$

H_{Mean} defines the mean height of the vertical niche relative to the maximum height of a standardized forest, and thus may vary between 0 and 1. The breadth of the vertical niche H_{Range} is randomly chosen from the defined ranges H_{Range}:

$$H_{Range} = \mathrm{unif}({H_{Range}}^{L}, {H_{Range}}^{U}) \tag{18}$$

The lower and upper boundary of the vertical niche can be determined based on H_{Mean} and H_{Range} as follows:

$$H_{Low} = \max\left(0, H_{Mean} - \frac{H_{Range}}{2}\right) \tag{19}$$

$$H_{Up} = \min\left(1, H_{Mean} + \frac{H_{Range}}{2}\right) \tag{20}$$

H_{Low} and H_{Up} are thus truncated when exceeding the natural vertical limits of 0 and 1. Based on the upper and lower limits of the vertical niche, the limits of the light niche are estimated as follows:

$$I_{Low} = I_{Max} \cdot e^{-(k_L \cdot LAI * (1-H_{Low}))} \tag{21}$$

$$I_{Up} = I_{Max} \cdot e^{-(k_L \cdot LAI * (1-H_{Up}))} \tag{22}$$

where I_{Max} is the average light intensity above the canopy and the LAI is the leaf area index in the hypothesized, standardized forest. We assume that the light optimum of each species lies midway between I_{Min} and I_{Max}:

$$I_{Opt} = \frac{I_{Low} + I_{Up}}{2} \tag{23}$$

I_{Low} and I_{Up} define the light intensities under which a species can survive. Please note that our approach of first specifying the height niche and translating it to the light niche may overestimate the values of I_{Low} for some species occurring in the outer canopy. These epiphyte species are commonly small, enabling them to occur on tiny branches, and have a range of adaptions to survive in the dry and nutrient-poor environment. They might well survive slightly more shaded conditions, but due to their other adaptations they mainly occur in the very upper canopy and have a rather narrow height niche. In our approach, where we directly translate these height niches to light niches without considering other traits and adaptations, the minimum light conditions for survival I_{Low} might hence be overestimated.

It would be too simplistic to assume that the growth of a species would be constant under all light conditions in the niche. Thus, we assumed that growth would be maximal under optimal light conditions I_{Opt}, and is reduced when deviating from this optimum. We use a parabolic growth response to simulate this situation (see *Growth* for more details), whereby the vertex of the parabola is given by I_{Opt}. I_{Low} and I_{Up} define the points at which the growth response becomes zero.

The parameters of this growth response function (I_a, I_b *and* I_c) are thus species-specific parameters that are calculated as follows:

$$I_a = \frac{I_{Up} - I_{Low}}{(I_{Low} - I_{Up}) \cdot (I_{Low} - I_{Opt}) \cdot (I_{Opt} - I_{Up})} \tag{24}$$

$$I_b = \frac{{I_{Up}}^2 - {I_{Low}}^2}{(I_{Low} - I_{Up}) \cdot (I_{Low} - I_{Opt}) \cdot (I_{Up} - I_{Opt})} \tag{25}$$

$$I_c = \frac{({I_{Low}}^2 \cdot I_{Up}) - (I_{Low} \cdot {I_{Up}}^2)}{(I_{Low} - I_{Up}) \cdot (I_{Low} - I_{Opt}) \cdot (I_{Up} - I_{Opt})} \tag{26}$$

Now, the species-specific parameters related to dispersal and reproduction are defined. We use a negative exponential function to describe the dispersal kernel (see *Establishment*), and the species-specific parameter D_K describing the shape of the kernel is randomly chosen from the uniform distribution on the interval [${D_K}^L$, ${D_K}^U$]:

$$D_K = \text{unif}\left({D_K}^L, {D_K}^U\right) \tag{27}$$

Dispersal kernels define the probability of dispersal as a function of distance from the source. They are mainly use in one- or two-dimensional space. A simple application of common kernels in three-dimensional space might not be adequate, as due to the effect of gravity downward dispersal is more likely than upward dispersal. To account for this effect, we additionally defined the species-specific dispersal kernel asymmetry D_{KAs}, which is randomly chosen as follows:

$$D_{KAs} = \text{unif}\left({D_{KAs}}^L, {D_{KAs}}^U\right) \tag{28}$$

This traits describes the probability that seeds are dispersed below the mother plant; hence D_{KAs}=0.5 describes a symmetric dispersal in all direction (for more details see *Establishment*).

Finally, traits related to the fecundity of the species are defined, and the potential average number of recruits per mature plant n_{RPot} is randomly chosen based on the defined ranges as follows:

$$n_{RPot} = \text{unif}({n_{RPot}}^{L}, {n_{RPot}}^{U}) \qquad (29)$$

This model does not separate the processes seed dispersal, germination and seedling establishment; n_{RPot} can thus be understood as average number of seedlings from a single mother plant that could establish in one year if substrate area of 1 m^2 per voxel would be available in the surroundings of the mother (for more details see *Establishment*).

Generation of initial distribution

Based on the local species set, an initial spatial distribution of the epiphyte assemblage is generated. First, the number of individuals per species and ha (n_{Ha}) and the ratio of juvenile to mature plants (r_{MJ}) are defined. Subsequently, a list containing all individuals in the assemblage is generated, and individuals of each species are divided into juveniles/adults according to r_{MJ}. For each juvenile, the initial body mass M is randomly chosen as follows:

$$M = \text{unif}\left(\frac{M_{Mat}}{100}, M_{Mat}\right) \qquad (30)$$

The initial mass of each adult is estimated accordingly:

$$M = \text{unif}(M_{Mat}, M_{Max}) \qquad (31)$$

The positions in the list containing all individuals are randomly shuffled, and following this order each individual is distributed on the initial microhabitat matrix. Specifically, this means that, at first, all voxels having light conditions within the species-specific light niche (I_{Low}, I_{Up}) are selected as

potential habitat. Subsequently, voxels whose unoccupied surface area is smaller than the space requirements of the individual are excluded. The space requirements are calculated as follows:

$$S = M^{2/3} \cdot g_S \qquad (32)$$

where g_S is a scaling parameter. This means, we assume that the space occupied by an individual scales with its mass to the power 2/3, and g_S relates $M^{2/3}$ to occupied surface area. Finally, after all potential voxels have been identified, a single one is randomly selected as initial location of the individual and the total occupied surface area in this voxel is updated accordingly. This procedure is repeated for all individuals. If there should be no suitable voxel for an individual, this is recorded and thus allows evaluating the adequacy of the initial distribution before starting the actual simulation.

Recruitment

Dispersal and recruitment are simulated together as one process. In this process, the number of new recruits and their spatial position is calculated, which is mainly determined by the species-specific fecundity (described by the potential average number of recruits per individual n_{RPot}), the species-specific dispersal ability D_{K} and the dispersal kernel asymmetry D_{KAs}, as well as by the available substrate (tree and branch surface area) in the surroundings of the mature epiphyte individual. n_{RPot} describes the potential average number of recruits if a substrate area of 1 m^2 were available in each voxel (1 m^3) surrounding the mature epiphyte, with D_{K} and D_{KAs} determining which voxels have which probability for recruitment. As the substrate area in forests is not evenly distributed and in most areas much lower than 1 m^2 per 1 m^3, the effective number of recruits is usually substantially lower than n_{RPot} and in areas with low substrate density, there may be years in which some individual may not produce a single recruit. A technical description of the recruitment process is given in the following.

Recruitment in each time step is based on 3D probability matrices. To calculate these matrices, 3D distance matrices, whose size in X, Y and Z direction is twice as large (plus 1) as that of the

microhabitat matrix, are first calculated. The Euclidian distance to the center is calculated for each voxel of these matrices. Based on the distances, the probability for dispersal in each voxel is calculated using the dispersal kernel which is described by a negative exponential function:

$$P_{DR} = \mathrm{e}^{-Dist_V \cdot D_K} \quad (33)$$

where $Dist_V$ is the distance to the central voxel and D_K is the species-specific dispersal trait. We assume that the dispersal kernel is not symmetric in Z direction, and the species-specific asymmetry is defined by D_{KA}. The probabilities of dispersal in all voxel above the central voxel are thus modified as follows:

$$P_D = P_{DR} \cdot 2 \cdot (1 - D_{KA}) \quad (34)$$

Accordingly, the probabilities of dispersal in all voxel below the central voxel are modified:

$$P_D = P_{DR} \cdot 2 \cdot D_{KA} \quad (35)$$

After P_D for all voxels are calculated, the probabilities are normalized so that: $\sum P_D=1$.

Subsequently, for each adult, the probability matrix is multiplied with n_{RPot} (element-wise multiplication) and a sub-matrix of this probability matrix is generated. This sub-matrix has the same dimensions as the microhabitat matrix and is selected in such a way that the 3D position of the individual epiphyte matches the central voxel of the probability matrix (This sub-setting step is the reason for the doubled dimensions of the probability matrix as this allows to generate an adequate subset at all possible position of epiphyte in the microhabitat matrix). This matrix is multiplied with the surface matrix (containing all S_V) and the niche matrix, which describes suitable voxel, based on the light intensity I^{XYZ} and the species-specific light traits (I_{Low}, I_{Opt}, I_{Up}), with a 1 and unsuitable ones with a 0. The resulting final matrix describes how many new recruit of a species could potentially

establish in each of the voxels of the microhabitat matrix. The actual number of new recruits is then estimated based on Poisson random values.

Growth

Growth of each individual is simulated as follows:

$$M_{(t+1)} = M_{(t0)} + k \cdot (M_{Max} - M_{(t0)}) \cdot (I_A \cdot (I^{XYZ})^2 + I_B \cdot I^{XYZ} + I_C) \quad (36)$$

This equation combines a von Bertalanffy growth function, in which the growth rate declines with increasing body mass, and a parabolic light-response function.

We opted for the von Bertalanffy growth function following two main conditions considered for a suitable growth function. On the one hand, the function should be an appropriate approximation of epiphyte growth in general. On the other hand, the function should be as simple as possible and its parameters should be identifiable within our functional trait framework. Note that we are not simulating real species for which growth data is available (which is rare for epiphytes), but rather artificial species for which a set of functional traits has to be defined in a generalizable fashion. In this case, growth parameters should hence be derivable from known traits. From the several possible plant growth functions (Paine et al., 2012), the von Bertalanffy function best fulfilled the conditions. It describes reasonably well the few known growth trajectories of epiphytes (Schmidt and Zotz, 2002; Zotz 1995), and it can be described by only one parameter which can be directly derived from three other traits (Eq. 16). Future model development may consider testing and implementing alternative growth functions, for instance when the dynamics of a single real-world species should be simulated in detail.

For epiphytes, it is yet unknown if any specific light response curve of growth is dominant, i.e. if a specific type (bell-shaped, parabolic, symmetrical, or asymmetrical) is most common across species. We thus chose a parabolic response because of its simplicity. We are simulating artificial species and

are mainly interested in community dynamics, and hence growth is not the most crucial process. We are certain that using a different light response would not significantly alter model results at the community level. However, future model development may consider implementing other alternative light response functions, particularly if the model is to be applied for selected epiphyte species for which growth patterns are known.

Mortality

First, the mortality due to branch or tree fall is simulated based on the relative change (loss) of surface in the voxel of the individual. We assume that the probability that an individual falls attached to a branch equals the relative loss rate of surface area. This means, if 30% of the area in a voxel is lost due to branch fall, the mortality probability is m_{BF}=0.3. For each individual, random numbers are drawn from the uniform distribution on the interval [0, 1] and, on this basis, mortality is determined. Please note that branch and tree fall in the forest model is simulated as a mixture of deterministic and stochastic processes. Branches fall when they are shaded for a long time, when neighboring trees fall, and to a certain extent by chance. Trees die when they cannot meet their metabolic demands, i.e. when the light conditions are below the species-specific threshold for a longer period, which results in increased mortality rates in smaller trees. In addition, a mass-dependent stochastic tree mortality following the metabolic theory is integrated.

Second, individuals die when light conditions are outside their light niches. This type of mortality thus occurs when the forest structure changes with time, accompanied with environmental (light) changes. For instance, when a large tree falls, the light intensities in the trees near the gap is increased, and the maximum light intensities for survival of shade specialists might be exceeded.

Third, we use a mortality rate following the MTE to account for cause of mortality not considered in this model. In this case, the mortality probability scales with the mass of an individual and is calculated as follows:

$$m_{MTE} = k_M + M^{-1/4} \qquad (37)$$

where k_M is the intercept of this scaling function. For each individual, random numbers are drawn from the uniform distribution on the interval [0, 1] and, on this basis, mortality is determined.

Fourth, mortality due to competition is simulated. If the total surface area in a voxel is lower than the space required by all epiphytes in the voxel, the smallest individual is removed successively until the space requirements are fulfilled. This procedure is only applied to voxel with at least two individuals. If a voxel contains a single individual that grows beyond the physical space present in the voxel, we assume that this individual also dies. Both processes are summarized as mortality due to competition.

Finally, all dead individuals are removed from the community, the age of all surviving ones are updated and the model proceeds with the next time step.

Table S2. List of abbreviations.

Symbol	Explanation	Unit
A	Age of epiphyte	year
A_{LTot}^{XYZ}	Total leaf area in voxel (per m^2)	$cm^2\ m^{-2}$
A_{Mat}	Age at maturity	year
A_{MatDev}	Relative deviation from mean age of maturity	%
C_R	Relative contribution of each ring (used in calculations of I^{XYZ})	-
$Dist_V$	Distance of a voxel to the centre voxel (distance matrix)	-
D_K	Dispersal ability - factor B in negative exponential function	-
D_{KAs}	Dispersal kernel asymmetry	-
D_S	Diameter of branch segment	cm
D_T	Diameter of trunk	cm
E_X, E_Y, E_Z	Position of epiphyte in model space in X, Y, Z direction	m
g_S	scaling factor relating epiphyte biomass to occupied area	-
H_{Low}	Lower boundary of the vertical niche of epiphyte species, relative to the maximum height of a standardized forest	-
H_{Mean}	Mean height of the vertical niche of epiphyte species, relative to the maximum height of a standardized forest	-
H_{Range}	Breadth of the vertical niche of epiphyte species, relative to the maximum height of a standardized forest	-
H_{Up}	Upper boundary of the vertical niche of epiphyte species, relative to the maximum height of a standardized forest	-
I_A, I_B, I_C	Parameter A, B, C of parabolic light response curve	-
ID_{Ind}	Individual ID of epiphyte	-
ID_{Sp}	Species ID of epiphyte	-
I_{Low}	Minimum light intensity for survival (lower boundary)	$\mu mol\ m^{-2}\ s^{-1}$
I_{max}	Light intensity above canopy	$\mu mol\ m^{-2}\ s^{-1}$
I_{Opt}	Optimum light intensity	$\mu mol\ m^{-2}\ s^{-1}$
I_{SC}^{XYZ}	Single column light intensity in voxel	$\mu mol\ m^{-2}\ s^{-1}$
I_{Up}	Maximum light intensity for survival (upper boundary)	$\mu mol\ m^{-2}\ s^{-1}$
I^{XYZ}	Effective light intensity in voxel	$\mu mol\ m^{-2}\ s^{-1}$
K	Growth rate (von Bertalanffy growth)	$year^{-1}$
k_L	Light extinction coefficient (Lambert-Beer equation)	-
k_M	Intercept of the scaling function that scales mortality with the mass of an individual (following MTE, scaling factor -0.25)	-
k_{Mat}	Intercept of the scaling function that scales age at maturity A_{Mat} with maximum mass M_{Max} (following MTE, scaling factor 0.25)	-
LAI^{XYZ}	Leaf area index for each voxel	-
LR	Number of rectangular rings around the focal voxel to be considered in light calculation	-
L_S	Length of branch segment	cm
L_T	Length of trunk	cm
L_{TS}	Length of trunk segment	cm
L_V	Side length of voxels	m
M	Mass of epiphyte	g
m_{BF}	Mortality probability due to substrate loss	-
M_{Mat}	Mass at maturity	g
M_{Max}	Maximum mass of species	g
m_{MTE}	Mortality probability following the MTE	-
M_{Rel}	Ratio between M_{Mat} and M_{Max}	-
n_{Ha}	Number of individuals per species and ha in initial distribution	-
n_{Int}	Total number of voxels intersecting with branch segments	-

n_{RPot}	Potential average number of recruits per individual	-
n_{SP}	Number of species in species set	-
P_{D}	Dispersal probability (considering the dispersal kernel asymmetry)	-
P_{DR}	Dispersal probability (without considering the dispersal kernel asymmetry)	-
$P_{\mathrm{SEnd}}^{\mathrm{XYZ}}$	End position of branch segment (in X, Y and Z direction)	cm
$P_{\mathrm{SStart}}^{\mathrm{XYZ}}$	Start position of branch segment (in X, Y and Z direction)	cm
$P_{\mathrm{T}}^{\mathrm{XY}}$	Position of trunk (in X and Y direction)	cm
R	Ring number (used in calculations of I^{XYZ})	-
r_{Int}	Radius at the intersection between trunk part and voxel	cm
r_{MJ}	Ratio of juvenile to mature plants in initial distribution	-
S	Space requirement of individual epiphyte	cm^2
S_B	Total surface area of branches in voxel	cm^2
S_{BS}	Surface area of branch segment	cm^2
S_{Loss}	Percentage annual surface area change in voxel	%
S_{V}	Total surface area of arboreal substrate in voxel	cm^2
V_{IntX}, V_{IntY}, V_{IntZ}	Voxels intersecting with branch segments in X, Y, Z direction	-
V_{X}, V_{Y}, V_{Z}	Position of voxel in model space in X, Y, Z direction	m
V_{ZMax}	Highest voxel a trunk is intersecting with	-

References

Brown, J. H., Gillooly, J. F., Allen, A. P., Savage, V. M., & West, G. B. (2004). Toward a metabolic theory of ecology. *Ecology*, 85(7) , 1771-1789.

Cabral, J. S., Petter, G., Mendieta-Leiva, G., Wagner, K., Zotz, G., & Kreft, H. (2015). Branchfall as a demographic filter for epiphyte communities: lessons from forest floor-based sampling. *PloS One*, 10(6), e0128019.

Duncan, R. P., Forsyth, D. M., & Hone, J. (2007). Testing the metabolic theory of ecology: Allometric scaling exponents in mammals. Ecology, 88(2), 324–333.

Grimm, V., Berger, U., Bastiansen, F., Eliassen, S., Ginot, V., Giske, J., … DeAngelis, D. L. (2006). A standard protocol for describing individual-based and agent-based models. *Ecological Modelling*, 198(1–2), 115–126.

Grimm, V., Berger, U., DeAngelis, D. L., Polhill, J. G., Giske, J., & Railsback, S. F. (2010). The ODD protocol: A review and first update. *Ecological Modelling*, 221(23), 2760–2768.

Krömer, T., Kessler, M., & Gradstein, S. R. (2007). Vertical stratification of vascular epiphytes in submontane and montane forest of the Bolivian Andes: the importance of the understory. *Plant Ecology*, 189(2), 261–278.

Paine, C. E. T., Marthews, T. R., Vogt, D. R., Purves, D., Rees, M., Hector, A., & Turnbull, L. A. (2012). How to fit nonlinear plant growth models and calculate growth rates: An update for ecologists. *Methods in Ecology and Evolution*, 3(2), 245–256.

Petter, G., Wagner, K., Wanek, W., Sánchez Delgado, E. J., Zotz, G., Cabral, J. S., & Kreft, H. (2016). Functional leaf traits of vascular epiphytes: Vertical trends within the forest, intra- and interspecific trait variability, and taxonomic signals. *Functional Ecology*, 30(2), 188–198.

Petter, G., Kreft, H., Ong, Y., Zotz, G., & Sarmento Cabral, J. (2020). Modeling the long-term dynamics of tropical forests: from leaf traits to whole-tree growth patterns. *bioRxiv*. doi:10.1101/2020.06.01.128256.

Quesada, C. A., Phillips, O. L., Schwarz, M., Czimczik, C. I., Baker, T. R., Patiño, S., … Lloyd, J. (2012). Basin-wide variations in Amazon forest structure and function are mediated by both soils and climate. *Biogeosciences*, 9(6), 2203–2246.

Schmidt, G., & Zotz, G. (2002). Inherently slow growth in two Caribbean epiphytic species: A demographic approach. *Journal of Vegetation Science*, 13(4), 527–534.

Spruch, L., Hellwig, J., Zotz, G., & Blasius, B. (2019). Modeling community assembly on growing habitat “islands”: a case study on trees and their vascular epiphyte communities. *Theoretical Ecology*, 1–17.

Taylor, A., & Burns, K. (2015). Epiphyte community development throughout tree ontogeny: an island ontogeny framework. *Journal of Vegetation Science*, 26, 902-910.

Wagner, K., Bogusch, W., & Zotz, G. (2013). The role of the regeneration niche for the vertical stratification of vascular epiphytes. *Journal of Tropical Ecology*, 29, 277-290.

Wright, S. J. (2005). Tropical forests in a changing environment. *Trends in Ecology and Evolution*, 20(10), 553–560.

Zotz, G. (1995). How fast does an epiphyte grow? *Selbyana*, 16(2), 150-154.
