# Supplementary material for: Agent‐based modeling of the effects of forest dynamics, selective logging, and fragment size on epiphyte communities
Source: Ecol Evol. 2021 Feb 28;11(6):2937–51. doi: 10.1002/ece3.7255 (PMC7981202; doi:10.1002/ece3.7255)
Supplement: Supplementary file 3 — Appendix S3 [file ECE3-11-2937-s003.pdf]

Appendix A3

Additional figures and tables

to

Agent-based modeling of the effects of forest dynamics, selective logging, and fragment size on epiphyte communities

Gunnar Petter, Gerhard Zotz, Holger Kreft, Juliano Sarmento Cabral

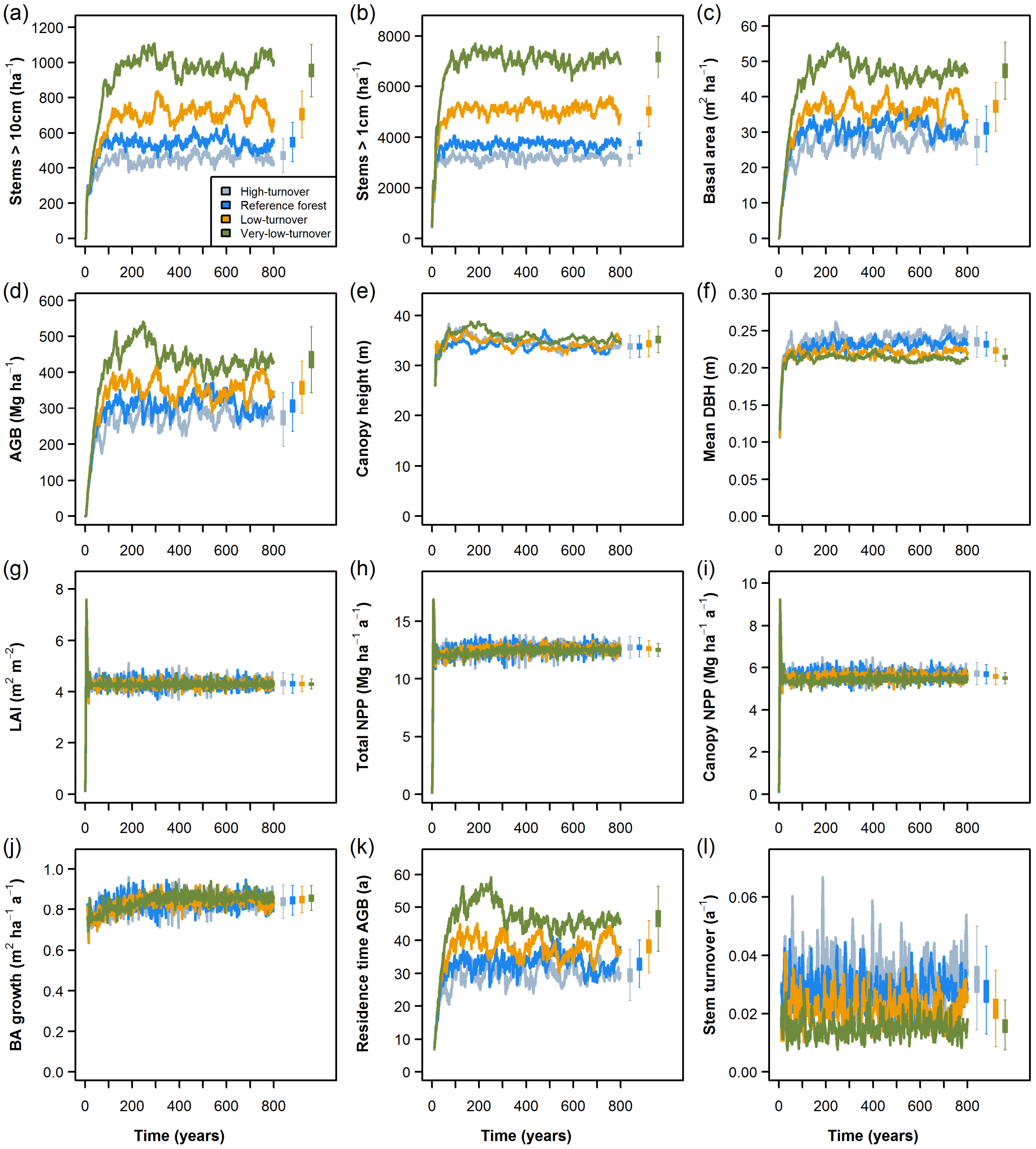

Figure S1. Long-term dynamics of the simulated forest stands differing in **natural forest dynamics.** Forest dynamics starting from bare ground are shown here. Please note that epiphyte simulations are initialized in year 200 on established forests, i.e. the epiphyte simulations from year 0-600 use the forest dynamics from year 200-800 as input forest data. For the static reference forest scenario (Fig. 4 in main manuscript), the reference forest in year 200 was used input data, i.e. the forest remained in this state for 600 years. (a) Stem density of all stems > 10 cm in DBH, (b) Stem density of all stems > 1 cm in DBH, (c) Basal area, (d) Above-ground biomass, (e) Canopy height (mean height of all trees > 40 cm in DBH; please note that the canopy height curve does not start at year 0 but rather between year 20-30 when the first tree reaches the threshold), (f) Mean diameter of all stems > 10 cm in DBH, (g) Leaf area index, (h) Total above-ground net primary production, (i) Canopy net primary production (NPP of leaves and 2nd order branches), (j) Basal area growth, (k) Residence time of above-ground biomass, (l) Turnover of all stems > 10 cm in DBH.

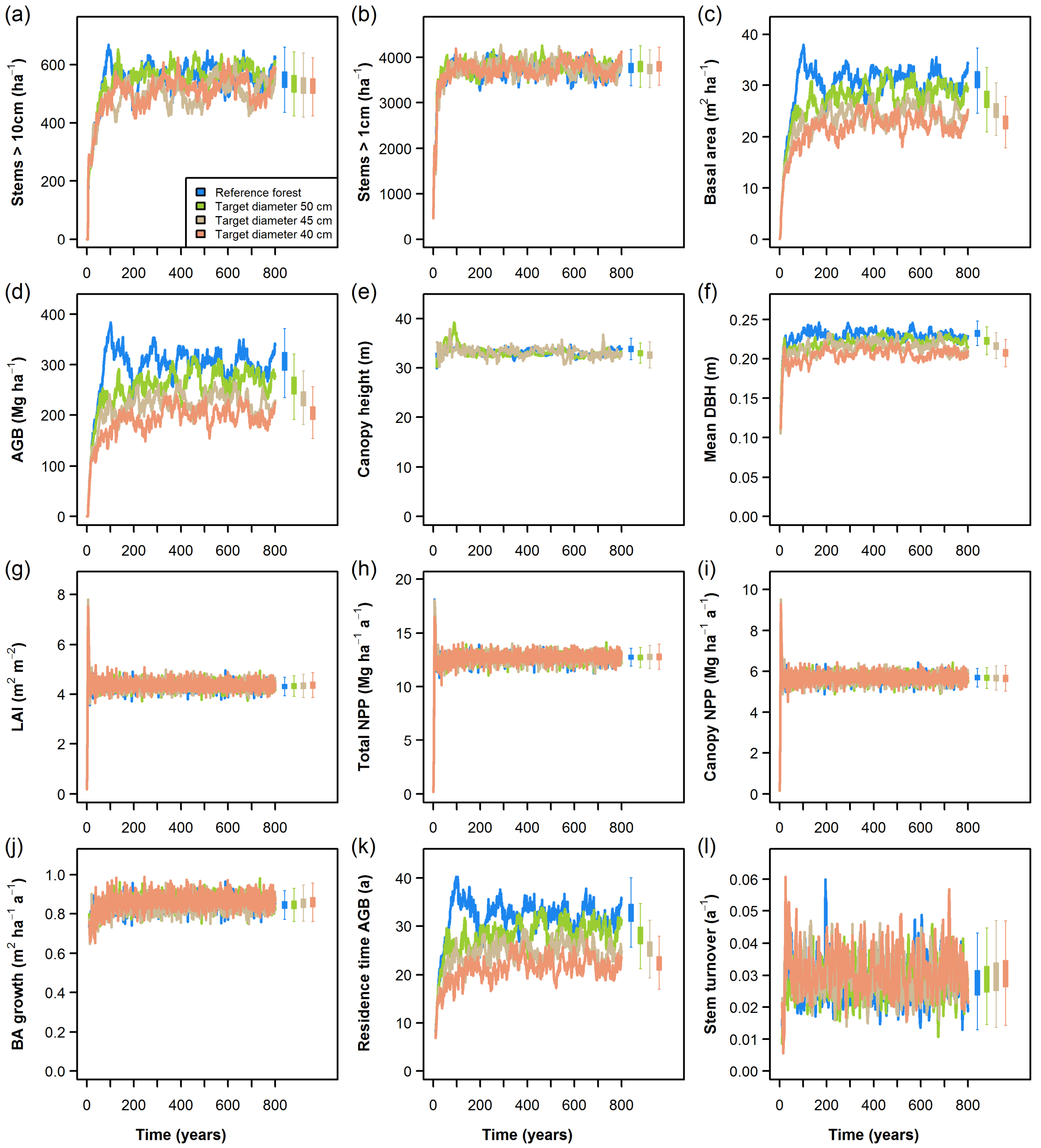

Figure S2. Long-term dynamics of simulated forest stands differing in the **target diameter for selective logging**. Forest dynamics starting from bare ground are shown here. Please note that epiphyte simulations are initialized in year 200 on established forests, i.e. the epiphyte simulations from year 0-600 use the forest dynamics from year 200-800 as input forest data. (a) Stem density of all stems > 10 cm in DBH, (b) Stem density of all stems > 1 cm in DBH, (c) Basal area, (d) Above-ground biomass, (e) Canopy height (mean height of all trees > 40 cm in DBH; please note that the canopy height curve does not start at year 0 but rather between year 20-30 when the first tree reaches the threshold), (f) Mean diameter of all stems > 10 cm in DBH, (g) Leaf area index, (h) Total above-ground net primary production, (i) Canopy net primary production (NPP of leaves and 2nd order branches), (j) Basal area growth, (k) Residence time of above-ground biomass, (l) Turnover of all stems > 10 cm in DBH.

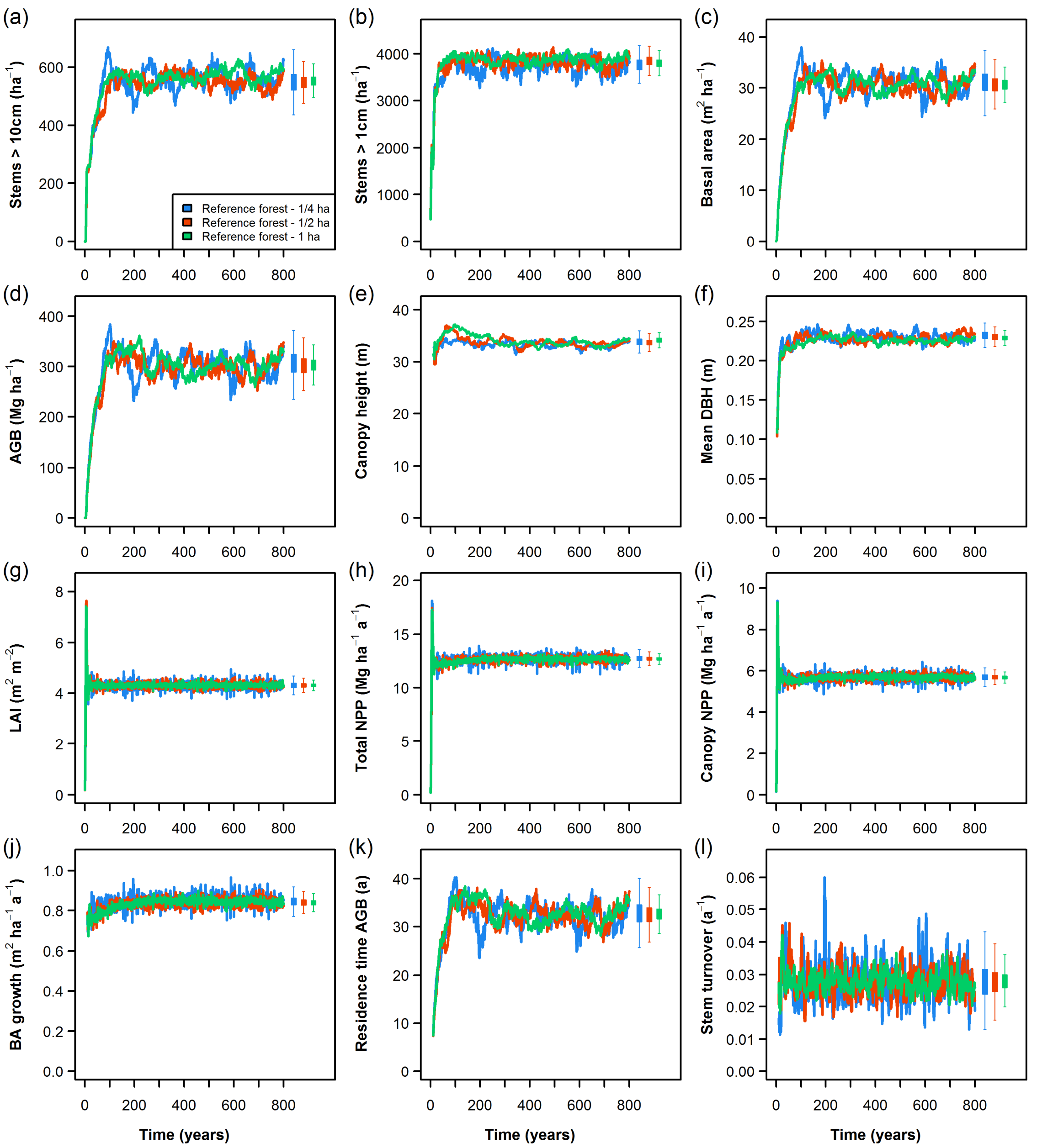

Figure S3. Long-term dynamics of simulated forest stands differing in **fragment size**. Forest dynamics starting from bare ground are shown here. Please note that epiphyte simulations are initialized in year 200 on established forests, i.e. the epiphyte simulations from year 0-600 use the forest dynamics from year 200-800 as input forest data. (a) Stem density of all stems > 10 cm in DBH, (b) Stem density of all stems > 1 cm in DBH, (c) Basal area, (d) Above-ground biomass, (e) Canopy height (mean height of all trees > 40 cm in DBH; please note that the canopy height curve does not start at year 0 but rather between year 20-30 when the first tree reaches the threshold), (f) Mean diameter of all stems > 10 cm in DBH, (g) Leaf area index, (h) Total above-ground net primary production, (i) Canopy net primary production (NPP of leaves and 2nd order branches), (j) Basal area growth, (k) Residence time of above-ground biomass, (l) Turnover of all stems > 10 cm in DBH.

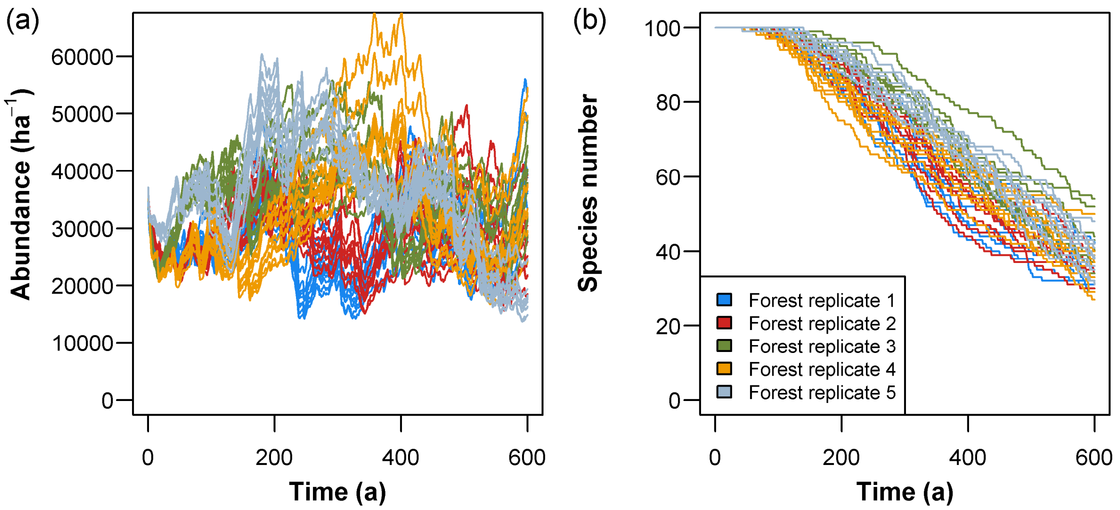

Figure S4. Simulated long-term dynamics of vascular epiphyte communities. Five replicates of a typical lowland forest stand (50 x 50 m, see Fig. S1 for forest attributes) were used as input data for the epiphyte model. On each of these forest replicates, the development of epiphyte communities, which initially consisted of 100 individuals of 100 species, was simulated over 600 years. Ten different initial species sets were simulated on each forest replicate and abundance (a) and species numbers (b) are shown here.

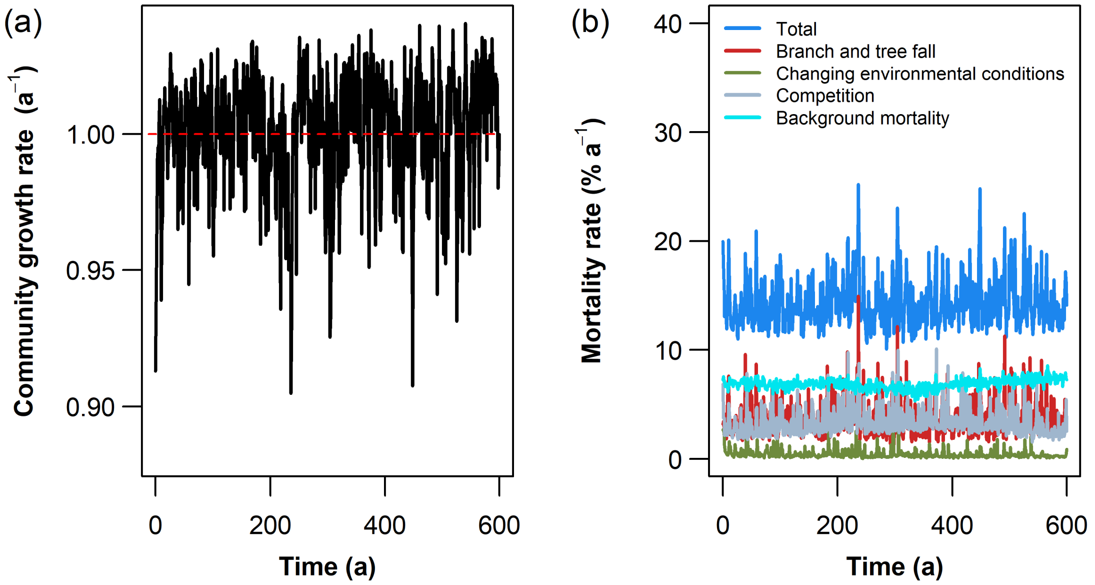

Figure S5. Community-wide growth rates and partitioning of mortality rates. Annual community growth rates and annual community-wide mortality rates of a representative epiphyte model run on the reference forest are shown to highlight annual fluctuation. See Table S2 for mortality rates averaged over all replicates.

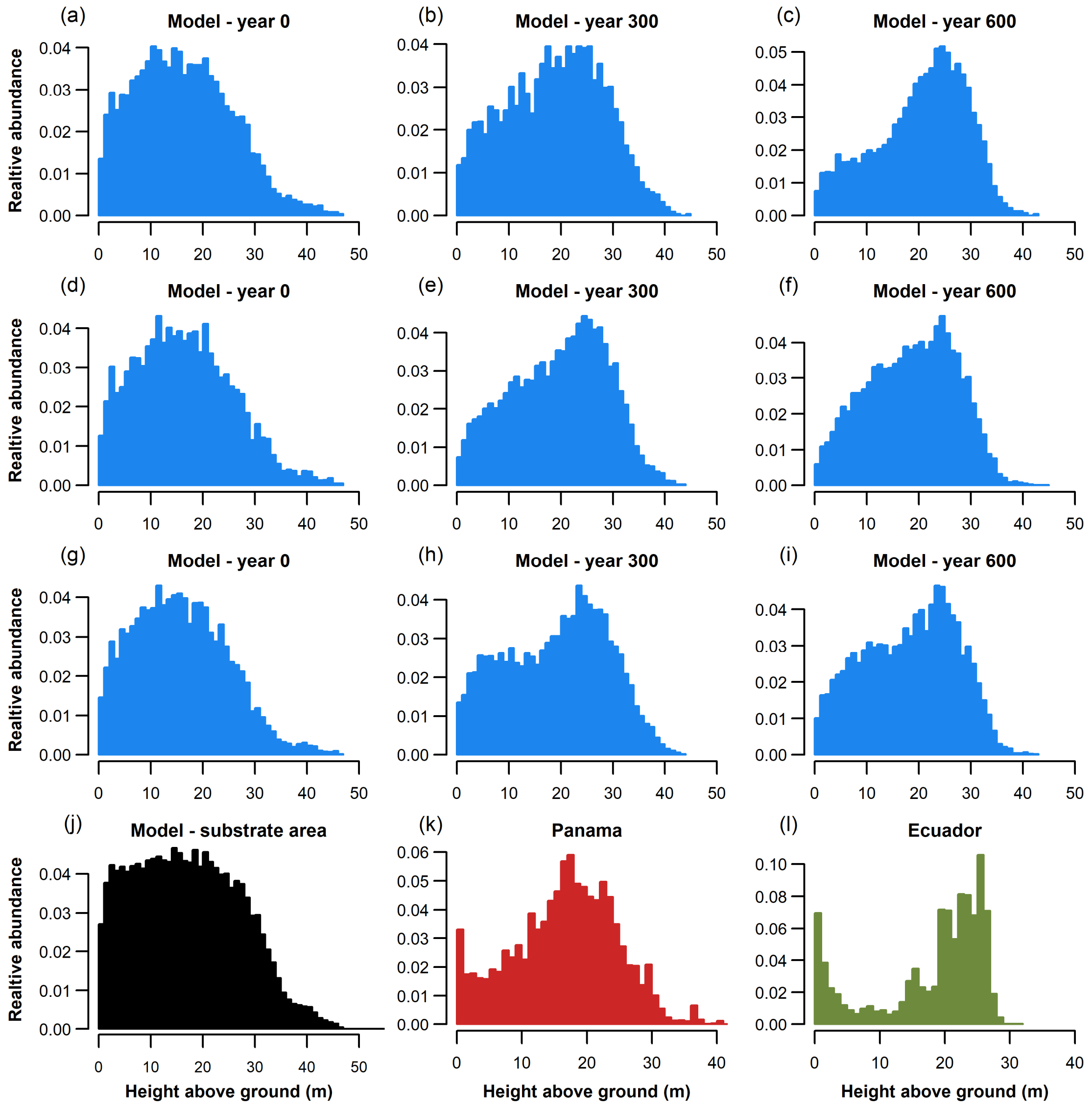

Figure S6. Vertical distribution of epiphytes. The panels a-i show the vertical distribution of 3 different epiphyte assemblages simulated on the reference forest at the beginning of the simulations (a,d,g), after 300 years (b,e,h) and after 600 years (c,f,i). The average vertical distribution of substrate area in theses forest stands is depicted in panel j. For comparisons: observed vertical distributions in Panama (k) and Ecuador (l).

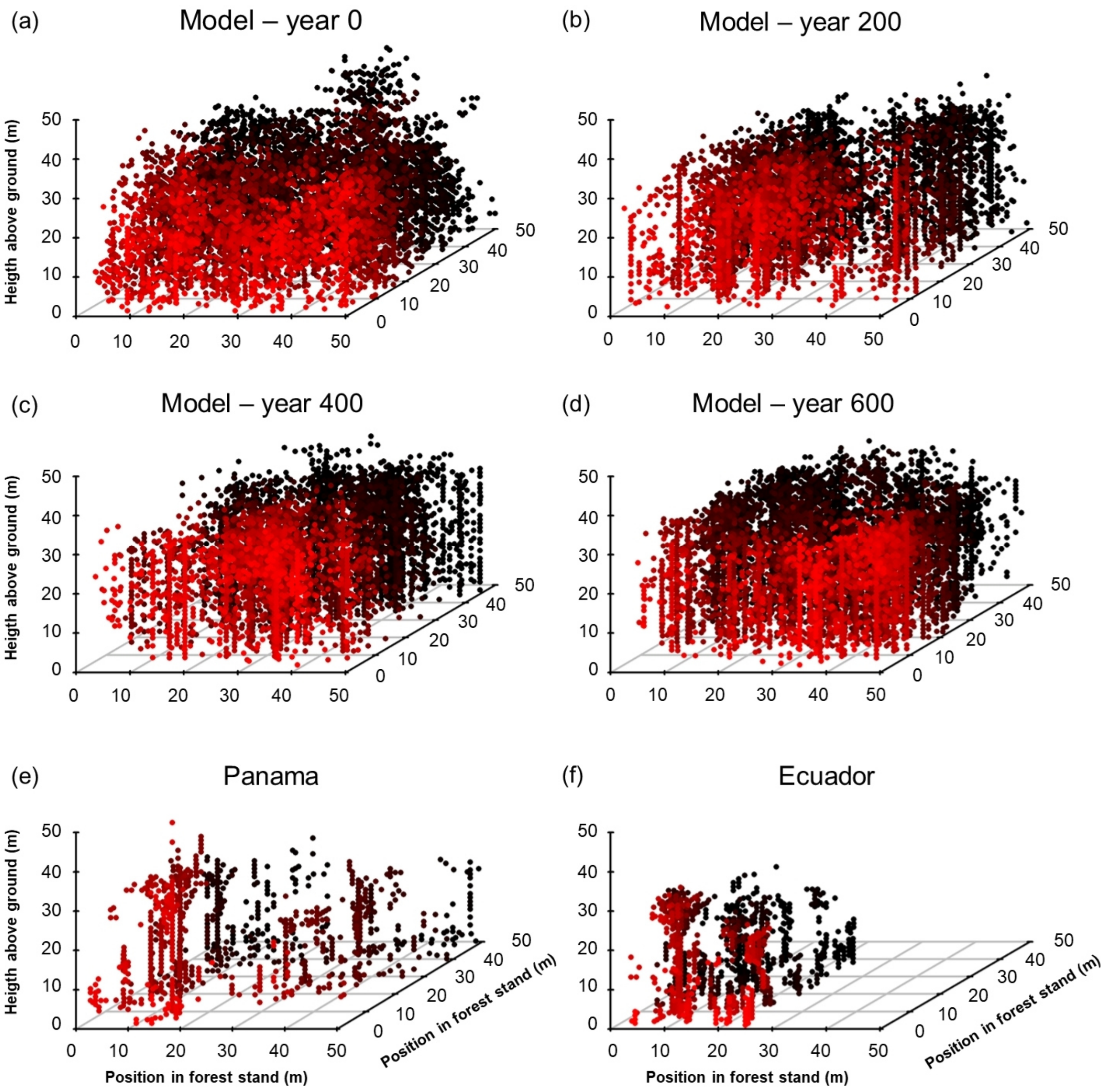

Figure S7. 3D structure of epiphyte assemblages. Panels a-d show the simulated epiphyte assemblage in comparison to field data from Panama (e) and Ecuador (f). The 3D positions of each epiphyte at year 0, 200, 400 and 600 in one representative model run on the reference forest are shown (a-d; see Fig. 2 in main manuscript for more details). The colors indicate the y-position, distant epiphytes are marked black, closer ones red. The epiphyte abundance at the shown time steps ranges from approximately 30.000 to 40.000 individuals per hectare. In Panama, epiphytes were sampled using a crane, and a section of the circular sampling area is shown (e). Due to the circular sampling area, not the entire 50x50m plot was sampled. In addition, a creek crosses the sampling area. In Ecuador, an area of 25x40 m was sampled (f). The incompletely sampled area is one of the reasons why the epiphyte density in Panama and Ecuador appears to be lower compared to the simulations, even though the total density in Panama is in a the same range (~40.000 ind. ha^{-1}), and even higher in Ecuador (~90.000 ind. ha^{-1}). In addition, the epiphyte distribution in Panama and Ecuador appears to be more clumped (we did not test this statistically).

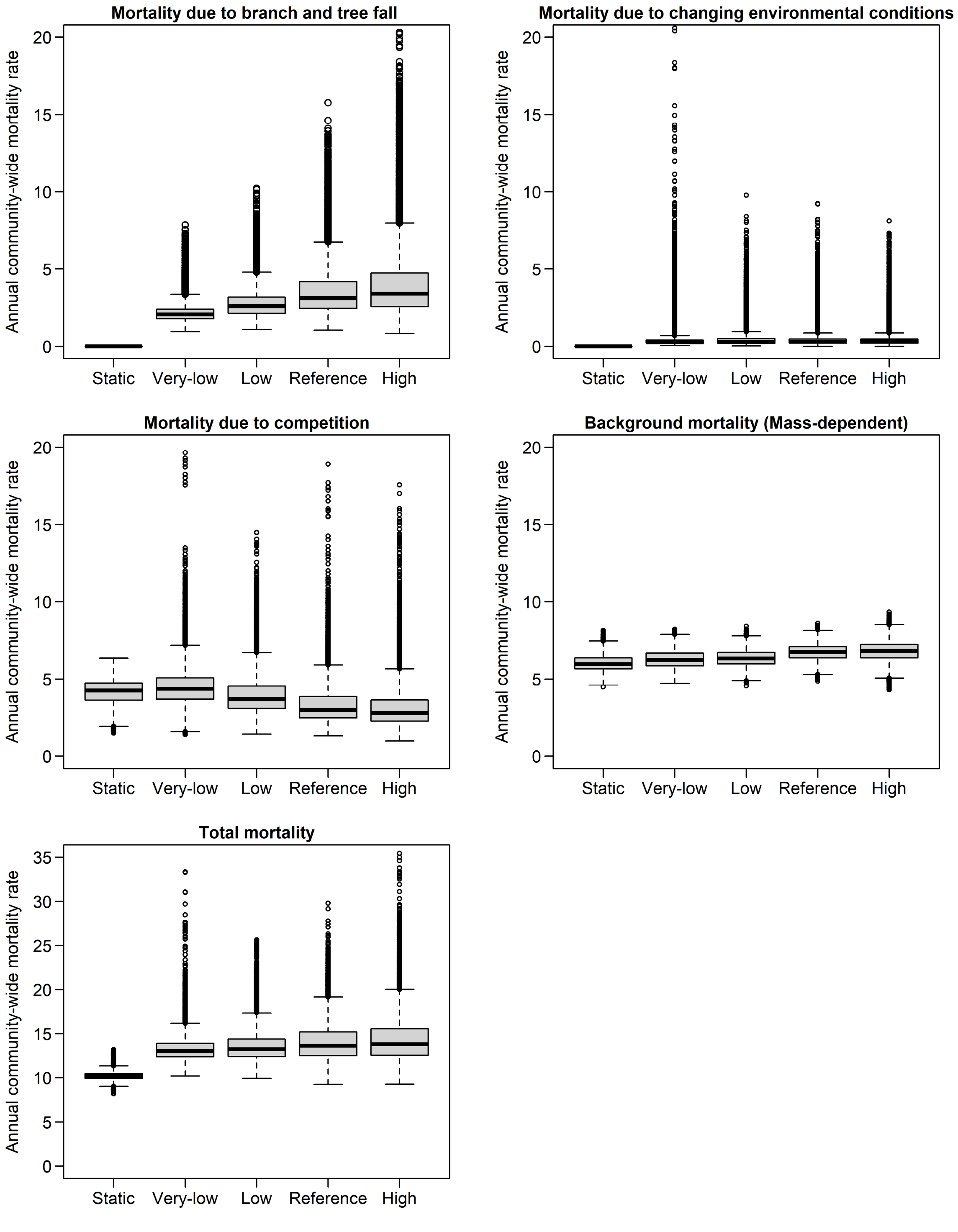

Figure S8. Mortality rates due to various causes in the forest dynamics scenarios. For each forest scenario, a total of 50 epiphyte simulations (five forest replicates x ten species sets) over 600 years were carried out. The annual community-wide mortality rates in all scenarios and time steps form the basis of the boxplots.

Table S1. Parameters and parameter ranges. The parameter values for global parameters and values ranges for epiphyte traits before and after model calibration are shown here. The value ranges after model calibration were calculated based on all species in the final species sets, comprising a total of 1000 species. In addition, mean ± standard deviation for these 1000 species are shown. Parameters used in the calibration and validation process are highlighted in bold. The initial value ranges were based on literature values or estimated based on our expert knowledge or the available datasets from Panama and Ecuador. For some unknown parameters, reasonable value ranges were estimated based on sensitivity analyses with the model.

Parameter	Description	Unit	Category	Value (range) before calibration	Value (range) after calibration	Mean±SD (calibrated)	Reference
A_{Mat}	Age at maturity	year	Epiphyte trait	[1.2, 18.5]	[2.0, 7.1]	3.7±1.0	Own estimate
D_K	Dispersal ability - factor B in negative exponential function	-	Epiphyte trait	[0.03, 0.50]	[0.24, 0.50]	0.41±0.06	Own estimate
D_{KAs}	Dispersal kernel asymmetry	-	Epiphyte trait	[0.50, 0.95]	[0.50, 0.95]	0.73±0.12	Own estimate
I_A	Parameter A of parabolic light response curve (derived from I_{Low}, I_{Up}, I_{Opt})	-	Epiphyte trait	[-0.88, -0000029]	[-0.00043, -0.000006]	-0.000039± 0.000050	Own estimate
I_B	Parameter B of parabolic light response curve (derived from I_{Low}, I_{Up}, I_{Opt})	-	Epiphyte trait	[0.00017, 5.07]	[0.0058, 0,0917]	0.0166±0.01 17	Own estimate
I_C	Parameter C of parabolic light response curve (derived from I_{Low}, I_{Up}, I_{Opt})	-	Epiphyte trait	[-54.51, -0.01035]	[-6.738, -0.384]	-1.38±1.13	Own estimate
I_{Low}	Minimum light intensity for survival	µmol m^{-2} s^{-1}	Epiphyte trait	[25, 687]	[25, 424]	114±79	Derived from relative height distribution
I_{Opt}	Optimum light intensity for survival	µmol m^{-2} s^{-1}	Epiphyte trait	[28, 793]	[97, 662]	353±156	Derived from relative height distribution
I_{Up}	Maximum light intensity for survival	µmol m^{-2} s^{-1}	Epiphyte trait	[32, 900]	[154, 900]	593±252	Derived from relative height distribution
K	Growth rate (Bertalanffy growth)	a^{-1}	Epiphyte trait	[0.028, 0.58]	[0.12, 0.52]	0.28±0.08	Schmidt & Zotz 2002; Zotz 1995
M_{Mat}	Mass at maturity	g	Epiphyte trait	[1, 2100]	[1, 107]	12±15	Own estimate
M_{Max}	Maximum mass	g	Epiphyte trait	[2, 3000]	[2, 175]	20± 24	Own estimate
**n_{RPot}**	**Average potential number of recruits per individual**	**-**	**Epiphyte trait**	**[1, 20]**	**[4.6, 7.5]**	**6.5±0.7**	Own estimate
A_{MatDev}	Relative deviation from mean age of maturity	%	Global parameter	25	25	-	Own estimate
**g_S**	**scaling factor relating epiphyte biomass to occupied area**	**-**	**Global parameter**	**[50, 200]**	**100**	-	Own estimate
I_{max}	Light intensity above canopy	µmol m^{-2} s^{-1}	Global parameter	900	900	-	Chazdon & Fetcher 1984; Berry, Varney & Flanagan 1997; Valladares, Allen & Pearcy 1997; Sterck *et al.* 2011; Seyoum *et al.* 2014
k_L	Light extinction coefficient (Lambert-Beer equation)	-	Global parameter	0.6	0.6	-	Huth & Ditzer 2000; Kitajima, Mulkey & Wright 2005; Malhi *et al.* 2013
**k_M**	**Intercept of the scaling function that scales mortality with the mass of an individual (following MTE, scaling factor -0.25)**	**-**	**Global parameter**	**[0.01, 1]**	**0.1**	-	Own estimate
**k_{Mat}**	**Intercept of the scaling function that scales age at maturity A_{Mat} with maximum mass M_{Max} (following MTE, scaling factor 0.25)**	**-**	**Global parameter**	**[1, 2.5]**	**2**	-	Own estimate
LAI	Leaf area index in standardized forest (used in generation of the species sets)	-	Global parameter	6	6	-	Myneni et al. 2007, Doughty & Goulden 2008, Caldararu, Palmer & Purves 2012

LR	Number of rectangular rings around the focal voxel to be considered in light calculation	-	Global parameter	8	8	-	Own estimate
n_{Ha}	Number of individuals per species and ha in initial distribution	-	Global parameter	400	400	-	Zotz & Schultz 2008
n_{Sp}	Number of species in species set	-	Global parameter	100	100	-	Zotz & Schultz 2008
r_{MJ}	Ratio of juvenile to mature plants in initial distribution	-	Global parameter	0.5	0.5	-	Own estimate

Table S2. Annual mortality rates in the reference forest scenario. For each individual that dies in the simulations, the cause of death is recorded. Annual community-wide mortality rates were estimated by dividing the total number of dead individual due to a specific cause by the total epiphyte abundance at the beginning of the year. Here, the mean, minimum and maximum annual mortality rates over the entire simulation period (600 years) and all replicates (5 forest replicates, 10 species sets) are shown.

Cause of mortality	Mean	Min	Max
Branch and tree fall	3.5	1.0	22.0
Changing environmental conditions	0.5	0.0	9.9
Competition	3.4	1.1	20.6
Background mortality (Mass-dependent)	6.7	4.8	9.3
Total	14.2	9.8	32.2

Table S3. Mean trait values of all species surviving (plain numbers) and of species that went locally extinct (bold numbers) during several scenario simulations (until year 600). The ratio of trait values of surviving species and species that went locally extinct is given in Table S4. For units see Table S1.

	Reference forest (1/4 ha)	Reference forest (1/2 ha)	Reference forest (1 ha)	Reference forest static	Very-low-turnover	Low-turnover	High-turnover	Target diameter 40 cm	Target diameter 45 cm	Target diameter 50 cm
Age at maturity	3.9	3.9	3.8	4.1	4.2	4.1	4	3.6	3.9	4
	**3.5**	**3.5**	**3.4**	**3.0**	**3.3**	**3.3**	**3.6**	**3.7**	**3.6**	**3.6**
Dispersal ability	0.4	0.39	0.39	0.41	0.41	0.4	0.4	0.4	0.38	0.39
	**0.41**	**0.42**	**0.43**	**0.39**	**0.40**	**0.41**	**0.41**	**0.41**	**0.41**	**0.41**
Dispersal kernel asymmetry	0.73	0.73	0.73	0.74	0.74	0.73	0.73	0.7	0.72	0.73
	**0.73**	**0.73**	**0.73**	**0.72**	**0.72**	**0.73**	**0.73**	**0.73**	**0.73**	**0.73**
Growth rate	0.26	0.26	0.27	0.24	0.24	0.25	0.26	0.28	0.26	0.26
	**0.29**	**0.29**	**0.29**	**0.32**	**0.30**	**0.30**	**0.28**	**0.27**	**0.28**	**0.28**
Height breadth	0.52	0.52	0.51	0.48	0.5	0.51	0.53	0.51	0.57	0.55
	**0.47**	**0.45**	**0.43**	**0.50**	**0.48**	**0.47**	**0.47**	**0.49**	**0.47**	**0.47**
Light breadth	501	509	510	454	475	475	537	457	478	496
	**465**	**443**	**407**	**514**	**481**	**481**	**458**	**478**	**478**	**472**
Mass at maturity	16.6	15.3	14.3	18.2	20.5	18.6	17.0	14.5	16.1	17.0
	**9.8**	**9.0**	**8.2**	**3.9**	**6.1**	**7.1**	**10.8**	**12.3**	**11.5**	**10.7**
Maximum light intensity	606	617	622	565	585	578	648	553	555	585
	**584**	**565**	**529**	**631**	**599**	**605**	**574**	**593**	**602**	**596**
Maximum mass	27.4	25.3	23.5	29.9	33.7	30.6	28	23.6	26.7	28.1
	**16.1**	**14.7**	**13.5**	**6.5**	**10.0**	**11.8**	**17.8**	**20.3**	**18.9**	**17.6**
Maximum relative height	0.86	0.87	0.87	0.84	0.84	0.84	0.89	0.84	0.84	0.85
	**0.85**	**0.84**	**0.82**	**0.88**	**0.86**	**0.86**	**0.84**	**0.85**	**0.86**	**0.85**
Mean relative height	0.62	0.62	0.63	0.61	0.61	0.6	0.64	0.59	0.56	0.59
	**0.63**	**0.62**	**0.61**	**0.64**	**0.63**	**0.64**	**0.62**	**0.62**	**0.64**	**0.63**
Minimum light intensity	106	108	112	111	110	103	111	97	77	89
	**120**	**122**	**121**	**117**	**118**	**125**	**116**	**115**	**124**	**124**
Minimum relative height	0.34	0.35	0.36	0.35	0.35	0.33	0.36	0.33	0.28	0.30
	**0.63**	**0.62**	**0.61**	**0.64**	**0.63**	**0.64**	**0.62**	**0.62**	**0.64**	**0.63**
Optimum light intensity	356	363	367	338	348	341	379	325	316	337
	**352**	**344**	**325**	**374**	**359**	**365**	**345**	**354**	**363**	**360**
Potential average number of recruits	6.59	6.61	6.56	6.62	6.70	6.66	6.56	6.35	6.62	6.59
	**6.40**	**6.31**	**6.29**	**6.21**	**6.30**	**6.31**	**6.44**	**6.47**	**6.44**	**6.43**

Table S4. Ratio of mean trait values of surviving species and species that went locally extinct. The mean trait values of surviving species (Table S3) were divided by the mean trait values of locally extinct species (Table S3), and the relative deviation in percent was calculated. Positive values indicate that mean trait values of surviving species were larger, negative values indicate that mean trait values of locally extinct species were larger. For units see Table S1.

	Reference forest (1/4 ha)	Reference forest (1/2 ha)	Reference forest (1 ha)	Reference forest static	Very-low-turnover	Low-turnover	High-turnover	Target diameter 40 cm	Target diameter 45 cm	Target diameter 50 cm
Age at maturity	12.4%	10.8%	10.9%	38.5%	26.9%	21.5%	10.5%	-1.5%	6.6%	10.9%
Dispersal ability	-2.6%	-6.7%	-7.9%	5.4%	1.5%	-0.4%	-2.5%	-2.0%	-8.0%	-4.6%
Dispersal kernel asymmetry	-0.7%	0.3%	0.4%	1.7%	2.2%	0.4%	-0.7%	-4.4%	-1.3%	-0.4%
Growth rate	-10.5%	-8.0%	-7.6%	-24.6%	-17.9%	-15.7%	-9.2%	2.4%	-6.6%	-9.3%
Light breadth	11.3%	14.5%	17.8%	-3.3%	3.8%	9.2%	11.4%	5.6%	20.7%	17.8%
Light breadth	7.9%	15.0%	25.4%	-11.7%	-1.2%	-1.2%	17.3%	-4.5%	-0.1%	5.2%
Mass at maturity	69.3%	70.5%	73.4%	368.0%	237.7%	159.8%	57.2%	17.7%	40.0%	58.3%
Maximum light intensity	3.8%	9.2%	17.7%	-10.4%	-2.3%	-4.5%	12.9%	-6.7%	-7.8%	-1.8%
Maximum mass	70.7%	71.9%	74.8%	360.2%	236.1%	159.2%	57.6%	15.8%	41.6%	60.2%
Maximum relative height	1.7%	3.5%	6.4%	-4.4%	-1.7%	-2.2%	5.5%	-1.3%	-1.7%	0.0%
Mean relative height	-1.7%	-0.2%	3.1%	-5.3%	-3.9%	-7.0%	3.5%	-5.3%	-11.2%	-7.2%
Minimum light intensity	-12.4%	-11.5%	-8.0%	-4.9%	-6.6%	-17.3%	-4.7%	-16.1%	-37.7%	-28.3%
Minimum relative height	-1.7%	-0.2%	3.1%	-5.3%	-3.9%	-7.0%	3.5%	-5.3%	-11.2%	-7.2%
Optimum light intensity	1.0%	5.5%	12.9%	-9.6%	-3.0%	-6.7%	9.9%	-8.2%	-12.9%	-6.4%
Potential average number of recruits	3.0%	4.7%	4.2%	6.7%	6.4%	5.5%	1.9%	-1.9%	2.8%	2.4%

References

Berry, S.C., Varney, G.T. & Flanagan, L.B. (1997). Leaf $\delta^{13}C$ in *Pinus resinosa* trees and understory plants: variation associated with light and CO_2 gradients. *Oecologia*, 109, 499–506.

Caldararu, S., Palmer, P.I. & Purves, D.W. (2012). Inferring Amazon leaf demography from satellite observations of leaf area index. *Biogeosciences*, 9, 1389–1405.

Chazdon, R.L. & Fetcher, N. (1984). Photosynthetic light environments in a lowland tropical rain forest in Costa Rica. *Journal of Ecology*, 72, 553–564.

Doughty, C.E. & Goulden, M.L. (2008). Seasonal patterns of tropical forest leaf area index and CO_2 exchange. *Journal of Geophysical Research*, 113, G00B06.

Huth, A. & Ditzer, T. (2000). Simulation of the growth of a lowland Dipterocarp rain forest with FORMIX3. *Ecological Modelling*, 134, 1–25.

Kitajima, K., Mulkey, S.S. & Wright, S.J. (2005). Variation in crown light utilization characteristics among tropical canopy trees. *Annals of Botany*, 95, 535–547.

Malhi, Y., Farfán Amézquita, F., Doughty, C.E., Silva-Espejo, J.E., Girardin, C.A.J., Metcalfe, D.B., Aragão, L.E.O.C., Huaraca-Quispe, L.P., Alzamora-Taype, I., Eguiluz-Mora, L., Marthews, T.R., Halladay, K., Quesada, C.A., Robertson, A.L., Fisher, J.B., Zaragoza-Castells, J., Rojas-Villagra, C.M., Pelaez-Tapia, Y., Salinas, N., Meir, P. & Phillips, O.L. (2013). The productivity, metabolism and carbon cycle of two lowland tropical forest plots in south-western Amazonia, Peru. *Plant Ecology & Diversity*, 7, 1–21.

Myneni, R.B., Yang, W., Nemani, R.R., Huete, A.R., Dickinson, R.E., Knyazikhin, Y., Didan, K., Fu, R., Negrón Juárez, R.I., Saatchi, S.S., Hashimoto, H., Ichii, K., Shabanov, N. V, Tan, B., Ratana, P., Privette, J.L., Morisette, J.T., Vermote, E.F., Roy, D.P., Wolfe, R.E., Friedl, M.A., Running, S.W., Votava, P., El-Saleous, N., Devadiga, S., Su, Y. & Salomonson, V.V. (2007). Large seasonal swings in leaf area of Amazon rainforests. *Proceedings of the National Academy of Sciences of the United States of America*, 104, 4820–4823.

Schmidt, G., & Zotz, G. (2002). Inherently slow growth in two Caribbean epiphytic species: A demographic approach. *Journal of Vegetation Science*, 13(4), 527–534.

Seyoum, F., Fetene, M., Strobl, S. & Beck, E. (2014). Daily and seasonal courses of gas exchange and niche partitioning among coexisting tree species in a tropical montane forest. *Flora*, 209, 191–200.

Sterck, F., Markesteijn, L., Schieving, F. & Poorter, L. (2011). Functional traits determine trade-offs and niches in a tropical forest community. *Proceedings of the National Academy of Sciences of the United States of America*, 108, 20627–20632.

Valladares, F., Allen, M.T. & Pearcy, R.W. (1997). Photosynthetic responses to dynamic light under field conditions in six tropical rainforest shrubs occuring along a light gradient. *Oecologia*, 111, 505–514.

Zotz, G. (1995). How fast does an epiphyte grow? *Selbyana*, 16(2), 150–154.

Zotz, G. & Schultz, S. (2008). The vascular epiphytes of a lowland forest in Panama - species composition and spatial structure. *Plant Ecology*, 195, 131–141.
